# An improved procedure for gene selection from microarray experiments using false discovery rate criterion

**DOI:** 10.1186/1471-2105-7-15

**Published:** 2006-01-11

**Authors:** James J Yang, Mark CK Yang

**Affiliations:** 1 Biostatistics and Research Epidemiology, Henry Ford Health Sciences Center, Detroit, Michigan, USA; 2 Department of Statistics, University of Florida, Gainesville, Florida, USA

## Abstract

**Background:**

A large number of genes usually show differential expressions in a microarray experiment with two types of tissues, and the *p*-values of a proper statistical test are often used to quantify the significance of these differences. The genes with small *p*-values are then picked as the genes responsible for the differences in the tissue RNA expressions. One key question is what should be the threshold to consider the *p*-values small. There is always a trade off between this threshold and the rate of false claims. Recent statistical literature shows that the false discovery rate (FDR) criterion is a powerful and reasonable criterion to pick those genes with differential expression. Moreover, the power of detection can be increased by knowing the number of non-differential expression genes. While this number is unknown in practice, there are methods to estimate it from data. The purpose of this paper is to present a new method of estimating this number and use it for the FDR procedure construction.

**Results:**

A combination of test functions is used to estimate the number of differentially expressed genes. Simulation study shows that the proposed method has a higher power to detect these genes than other existing methods, while still keeping the FDR under control. The improvement can be substantial if the proportion of true differentially expressed genes is large. This procedure has also been tested with good results using a real dataset.

**Conclusion:**

For a given expected FDR, the method proposed in this paper has better power to pick genes that show differentiation in their expression than two other well known methods.

## Background

The development of microarray technologies has created unparalleled opportunities to study the mechanism of disease, monitor disease expression and evaluate effective therapies. Because tens of thousands of genes are investigated simultaneously with a technology that has not yet been perfected, assessing uncertainty in the decision process relies on statistical modelling and theory. One key function of any statistical procedure is to control the rate of erroneous decisions or, in the microarray case, rate of false discovery of responsible genes.

The above concern can be illustrated as a multiple comparisons problem. Suppose we are interested in testing *g *parameters (*μ*_1_,..., *μ*_*g*_) = ***μ***. For each individual parameter *μ*_*j*_, the null hypothesis is *H*_0*j *_: *μ*_*j *_= 0 and the alternative hypothesis is *H*_1*j *_: *μ*_*j *_≠ 0. This *μ*_*j *_can be thought as the difference in mean expressions of the *j*th gene under two different conditions in a microarray experiment. A conventional method to test each hypothesis is to take a sample and then calculate its *p*-value from a proper statistical test. If the calculated *p*-value is less than a threshold determined by a testing significance level, then *H*_0*j *_is rejected. However, when many hypotheses are simultaneously performed, a multiple comparisons procedure (MCP) has to be used to control the error rate [1].

The traditional MCP controls the probability of making any error in multiple selections, i.e., controls the familywise error rate (FWER). It has been shown, however, that this type of procedure is extremely conservative when the number of hypotheses is large. Alternatively, Benjamini and Hochberg [2] proposed the measure of *false discovery rate *(FDR) for which the expected proportion of false discoveries is controlled. This procedure is based on the idea that one can tolerate more false discoveries if the number of tests is large. For example, 5 false discoveries out of 10 selections is probably too many while 5 false discoveries out of 100 selections should be acceptable. This is particularly useful in microarray analysis since a very large number of genes usually show differential expressions. Therefore, controlling the FDR can greatly increase the power of discovery.

Benjamini and Hochberg [3] proved that Simes' procedure [4] can be used to control expected FDR at *α *(0 <*α *< 1) when tests of true null hypotheses are either independent or positively dependent. More specifically, let *p*_(1) _≤ *p*_(2) _≤ ..., ≤ *p*_(*g*) _be the ordered *p*-values and *J *+ 1 be the smallest integer satisfying

p(J+1)>(J+1)×αg.     (1)
 MathType@MTEF@5@5@+=feaafiart1ev1aaatCvAUfKttLearuWrP9MDH5MBPbIqV92AaeXatLxBI9gBaebbnrfifHhDYfgasaacH8akY=wiFfYdH8Gipec8Eeeu0xXdbba9frFj0=OqFfea0dXdd9vqai=hGuQ8kuc9pgc9s8qqaq=dirpe0xb9q8qiLsFr0=vr0=vr0dc8meaabaqaciaacaGaaeqabaqabeGadaaakeaacqWGWbaCdaWgaaWcbaGaeiikaGIaemOsaOKaey4kaSIaeGymaeJaeiykaKcabeaakiabg6da+iabcIcaOiabdQeakjabgUcaRiabigdaXiabcMcaPiabgEna0oaalaaabaacciGae8xSdegabaGaem4zaCgaaiabc6caUiaaxMaacaWLjaWaaeWaaeaacqaIXaqmaiaawIcacaGLPaaaaaa@425A@

If *J *≥ 1, then rejecting *H*_(0*j*)_, *j *= 1,..., *J *ensures the expected FDR at *α*. We call this method the *BH procedure*.

Suppose the number of true nulls is *g*_0_, or the proportion is *π*_0 _= *g*_0_/*g*. Benjamini and Hochberg [3] proved that the expected FDR of BH procedure is less than or equal to g0g
 MathType@MTEF@5@5@+=feaafiart1ev1aaatCvAUfKttLearuWrP9MDH5MBPbIqV92AaeXatLxBI9gBaebbnrfifHhDYfgasaacH8akY=wiFfYdH8Gipec8Eeeu0xXdbba9frFj0=OqFfea0dXdd9vqai=hGuQ8kuc9pgc9s8qqaq=dirpe0xb9q8qiLsFr0=vr0=vr0dc8meaabaqaciaacaGaaeqabaqabeGadaaakeaadaWcaaqaaiabdEgaNnaaBaaaleaacqaIWaamaeqaaaGcbaGaem4zaCgaaaaa@308E@*α*. When *g*_0 _is small compared to *g*, using the original *α *in (1) loses of power because it can be replaced by the larger value *α*/*π*_0 _and still control the FDP at *α*. Here, the power of a selection procedure is defined as the proportion of the alternative hypotheses that are correctly identified. Obviously, if we knew *π*_0 _in advance, we could replace *α *in (1) by *α*/*π*_0 _to increase power. Since *π*_0 _is unknown, the key question here is how to estimate the number of true null hypotheses *g*_0_.

In an earlier published paper, Benjamini and Hochberg [5] proposed an *adaptive procedure *(aBH) which, by simulation, showed a higher power than BH procedure. The idea of the aBH procedure is to estimate *g*_0 _based on the fact that the *p*-value under the alternative hypothesis is stochastically smaller than that under the null, which is uniformly distributed on (0, 1). On a quantile plot of *p*-values (*p*_(*j*) _versus *j*), the slope passing through (*j*, *p*_(*j*)_) and (*g *+ 1,1) increases just as *j *increases if the *P*_(*j*)_s are corresponding to the subset of true alternative hypotheses. The first time the slope decreases indicates a change point. Using this stopping rule in conjunction with the Lowest SLope (LSL) estimator, (1 - *p*_(*j*)_)/(*g *+ 1 - *j*), Benjamini and Hochberg proposed their aBH method to estimate *g*_0 _as follows:

### Alg. 1 aBH method in *g*_0 _estimation

1. If there is no hypothesis rejected by the BH procedure, then stop and declare no discovery.

2. Calculate *m*_(*j*) _= (1 - *p*_(*j*)_)/(*g *+ 1 - *j*), ∀*j *= 1,..., *g*.

3. Let J* be the largest value such that *m*_(J*) _<*m*_(J* - 1)_. Define *b *as the smallest integer larger than 1/*m*_(J*)_.

4. The number of true null hypotheses is estimated as g^0
 MathType@MTEF@5@5@+=feaafiart1ev1aaatCvAUfKttLearuWrP9MDH5MBPbIqV92AaeXatLxBI9gBaebbnrfifHhDYfgasaacH8akY=wiFfYdH8Gipec8Eeeu0xXdbba9frFj0=OqFfea0dXdd9vqai=hGuQ8kuc9pgc9s8qqaq=dirpe0xb9q8qiLsFr0=vr0=vr0dc8meaabaqaciaacaGaaeqabaqabeGadaaakeaacuWGNbWzgaqcamaaBaaaleaacqaIWaamaeqaaaaa@2F2D@ = min(*b*, *g*).

Recently, Storey and Tibshirani [6] observed and demonstrated in their Figure 1 that the density histogram of *p*-values based on *p*_1_,..., *p*_*g *_from a microarray experiment looked flat in the region of (0.5, 1). Based on the fact that the null *p*-values of genes are uniformly distributed, most of genes in the region of (0.5, 1) should be from the null. Therefore, they used a smoothing method to estimate *g*_0 _based on the flat region of the observed *p*-values. However, the implicit requirements of their method are: 1) *g *should be large, and 2) the proportion of true null hypotheses should also be large, such as 0.5, so that *g*_0 _can be estimated accurately. The method developed in this paper tries to bypass these two requirements so that a broad range of multiple testing problems can be applied. It is conceivable that when the number of relevant genes are known for certain target disease, a chip with a small number of genes will be widely used for diagnosis. The FDR methods, including both the BH and the aBH versions, have now been widely accepted for microarray analyses. They have been implemented in the R program [7], which can be incorporated into the Bioconductor package [8]. Several microarray analysis computer programs, such as SAM (Significance Analysis of Microarrays) [9] and GeneSpring [10] also use the FDR criterion to identify differentially expressed genes. Yang *et al*. [11] have used the FDR criterion to determine the sample size in microarray experiments.

## Methods

Without loss of generality, we describe the method in a one-sample testing problem. Its extension to commonly used paired-*t *test, two-sample *t *test, or nonparametric rank tests in microarray analysis is very straightforward. Let ***Y***_*i *_= (*Y*_*i*1_,..., *Y*_*ig*_)', *i *= 1,..., *n *be *g*-variate vector samples from populations with means ***μ ***= (*μ*_1_,..., *μ*_*g*_)'. Define ***Y***^(*j*) ^= (*Y*_1*j*_,..., *y*_*nj*_), *,j *= 1,..., *g *as a row vector for the *j*th component. The null hypothesis of the *j*th component is *H*_0*j *_: *μ*_*j *_= 0 and the alternative hypothesis is *H*_1*j *_: *μ*_*j *_≠ 0. Let the *p*-value for rejecting the null *H*_0*j *_be *p*_*j *_derived from a properly chosen test statistic.

Once the *p*-values are derived, we first summarize the information content of *p*-values using a differentiable real-valued decreasing function *h*, i.e., *h*(*p*_1_,..., *p*_*g*_) is decreasing to all its arguments. We call *h *the combining function and its value, *η *= *h*(*p*_1_,..., *p*_*g*_), the global statistic. Next, define *η*^(0) ^to be the observed global statistic of *η *derived directly from the observed data. Note that a small value of *p*_*j *_indicates *H*_0*j *_is less likely to be true. Since *h *is a decreasing function of its argument *p*_*j*_, a large value of *η*^(0) ^indicates a subset of the null hypotheses is likely to be true alternatives. To determine whether *η*^(0) ^is large enough to make such a claim, we need to know the distribution of *η *when all the nulls are true. The distribution of *η *depends on the distribution of its arguments, the correlation between its arguments, and the combining function *h*. In a microarray experiment, there seems no way to model or estimate so many correlations (see, for example, Chapter 6 of Pesarin [12]). Hence, a reasonable approach which can tackle the correlation within each experimental unit is to determine the critical value by a permutation test. More specifically, we calculate all *B *= 2^*n *^values of *η *based on (*ω*_1_***Y***_1_,..., *ω*_*n*_***Y***_*n*_), where *ω*_*i *_= ± 1 (The multivariate two-sample permutation method can be found in Pesarin [12]). Hence, we have a set of reference values {η1(0),…,ηB(0)}
 MathType@MTEF@5@5@+=feaafiart1ev1aaatCvAUfKttLearuWrP9MDH5MBPbIqV92AaeXatLxBI9gBaebbnrfifHhDYfgasaacH8akY=wiFfYdH8Gipec8Eeeu0xXdbba9frFj0=OqFfea0dXdd9vqai=hGuQ8kuc9pgc9s8qqaq=dirpe0xb9q8qiLsFr0=vr0=vr0dc8meaabaqaciaacaGaaeqabaqabeGadaaakeaacqGG7bWEiiGacqWF3oaAdaqhaaWcbaGaeGymaedabaGaeiikaGIaeGimaaJaeiykaKcaaOGaeiilaWIaeSOjGSKaeiilaWIae83TdG2aa0baaSqaaiabdkeacbqaaiabcIcaOiabicdaWiabcMcaPaaakiabc2ha9baa@3D93@ from the *B *permutations, and can now define the *p*-value of the global *η*^(0)^-statistic as

p(0)=∑b=1BI(ηb(0)≥η(0))B     (2)
 MathType@MTEF@5@5@+=feaafiart1ev1aaatCvAUfKttLearuWrP9MDH5MBPbIqV92AaeXatLxBI9gBaebbnrfifHhDYfgasaacH8akY=wiFfYdH8Gipec8Eeeu0xXdbba9frFj0=OqFfea0dXdd9vqai=hGuQ8kuc9pgc9s8qqaq=dirpe0xb9q8qiLsFr0=vr0=vr0dc8meaabaqaciaacaGaaeqabaqabeGadaaakeaacqWGWbaCdaahaaWcbeqaaiabcIcaOiabicdaWiabcMcaPaaakiabg2da9maalaaabaWaaabmaeaacqWGjbqsdaqadaqaaGGaciab=D7aOnaaDaaaleaacqWGIbGyaeaacqGGOaakcqaIWaamcqGGPaqkaaGccqGHLjYScqWF3oaAdaahaaWcbeqaaiabcIcaOiabicdaWiabcMcaPaaaaOGaayjkaiaawMcaaaWcbaGaemOyaiMaeyypa0JaeGymaedabaGaemOqaieaniabggHiLdaakeaacqWGcbGqaaGaaCzcaiaaxMaadaqadaqaaiabikdaYaGaayjkaiaawMcaaaaa@4BE9@

where *I*(*x*) is the indicator function that takes value 1 if the statement *x *is true and 0 if it is not. To determine whether the global null hypothesis of all *μ*_(*j*)_, *j *= 1,..., *g*, is zero is to evaluate if the global *p*-value, *p*^(0)^, is less than or equal to a significance level. However, this level is part of the estimation process. It will be determined by the data (see later Alg. 2).

When the global null hypothesis is rejected, it indicates that not all null hypotheses *H*_0*j*_s are true. Immediate question is, which subset of hypotheses is the true alternative. To determine whether to reject the hypothesis *H*_(0*j*) _is a multiple testing problem. We can, however, determine the size of true alternatives using the following iterated process. We regard the gene with the smallest *p*-value as the major contributor for the rejection and claim that the null hypothesis is not true with this gene. When this gene is removed from the data set, we continue the same process with the rest *g *- 1 genes and the *p*^(0) ^computed by (2) is now denoted by *p*^(1)^. The whole process is then repeated to produce a sequence of pseudo-global *p*-values, *p*^(*s*)^, *s *= 1,..., *g *- 1. We call the later step global *p*-value pseudo because it is only based on the subset of the original data. The detail of how to generate pseudo-global *p*-values is given in the **Algorithm **section. First, we will use these pseudo-global *p*-values to estimate the number of true null genes *g*_0_.

We observe that, intuitively, *p*^(0) ^≤ *p*^(1) ^≤ ... ≤ *p*^(*g*-1)^, and this monotone increasing property will be proved in (J.1) of the **Justification **section. In addition , we will prove in (J.2) of the Justification section that if *r *pseudo-global *p*-values are less than a given value *β*(0 <*β *< 1), the estimator of the number of null genes is

g^0=g−r+β/(1−β)2     (3)
 MathType@MTEF@5@5@+=feaafiart1ev1aaatCvAUfKttLearuWrP9MDH5MBPbIqV92AaeXatLxBI9gBaebbnrfifHhDYfgasaacH8akY=wiFfYdH8Gipec8Eeeu0xXdbba9frFj0=OqFfea0dXdd9vqai=hGuQ8kuc9pgc9s8qqaq=dirpe0xb9q8qiLsFr0=vr0=vr0dc8meaabaqaciaacaGaaeqabaqabeGadaaakeaacuWGNbWzgaqcamaaBaaaleaacqaIWaamaeqaaOGaeyypa0Jaem4zaCMaeyOeI0IaemOCaiNaey4kaSccciGae8NSdiMaei4la8IaeiikaGIaeGymaeJaeyOeI0Iae8NSdiMaeiykaKYaaWbaaSqabeaacqaIYaGmaaGccaWLjaGaaCzcamaabmaabaGaeG4mamdacaGLOaGaayzkaaaaaa@4173@

and this estimator ensures that *E *[g^0
 MathType@MTEF@5@5@+=feaafiart1ev1aaatCvAUfKttLearuWrP9MDH5MBPbIqV92AaeXatLxBI9gBaebbnrfifHhDYfgasaacH8akY=wiFfYdH8Gipec8Eeeu0xXdbba9frFj0=OqFfea0dXdd9vqai=hGuQ8kuc9pgc9s8qqaq=dirpe0xb9q8qiLsFr0=vr0=vr0dc8meaabaqaciaacaGaaeqabaqabeGadaaakeaacuWGNbWzgaqcamaaBaaaleaacqaIWaamaeqaaaaa@2F2D@] ≥ *g*_0_. (The conservativeness of this estimate under other conditions using other techniques has also been mentioned by Storey and Tibshirani [13] and Efron et al. [14].) Since the inequality *E *[g^0
 MathType@MTEF@5@5@+=feaafiart1ev1aaatCvAUfKttLearuWrP9MDH5MBPbIqV92AaeXatLxBI9gBaebbnrfifHhDYfgasaacH8akY=wiFfYdH8Gipec8Eeeu0xXdbba9frFj0=OqFfea0dXdd9vqai=hGuQ8kuc9pgc9s8qqaq=dirpe0xb9q8qiLsFr0=vr0=vr0dc8meaabaqaciaacaGaaeqabaqabeGadaaakeaacuWGNbWzgaqcamaaBaaaleaacqaIWaamaeqaaaaa@2F2D@] ≥ *g*_0 _holds for any value of *β*, the best choice of *β *is the one which makes g^0
 MathType@MTEF@5@5@+=feaafiart1ev1aaatCvAUfKttLearuWrP9MDH5MBPbIqV92AaeXatLxBI9gBaebbnrfifHhDYfgasaacH8akY=wiFfYdH8Gipec8Eeeu0xXdbba9frFj0=OqFfea0dXdd9vqai=hGuQ8kuc9pgc9s8qqaq=dirpe0xb9q8qiLsFr0=vr0=vr0dc8meaabaqaciaacaGaaeqabaqabeGadaaakeaacuWGNbWzgaqcamaaBaaaleaacqaIWaamaeqaaaaa@2F2D@ closest to *g*_0_. By defining Δ = *β*/(1 - *β*)^2 ^and *ρ*(*β*) = *r *- Δ, we observe that *ρ*(*β*) is an increasing function and then a decreasing function of *β *for the following reasons. When *β *is small, Δ increases slowly. However, *r *is an increasing function in *β *and *r *is always greater than 1. Therefore, *ρ*(*β*) is an increasing function for small value of *β*. On the other hand, when *β *→ 1, Δ reaches ∞. Since *r *is finite, *ρ*(*β*) becomes a decreasing function in this range. Since (3) is equivalent to g^0
 MathType@MTEF@5@5@+=feaafiart1ev1aaatCvAUfKttLearuWrP9MDH5MBPbIqV92AaeXatLxBI9gBaebbnrfifHhDYfgasaacH8akY=wiFfYdH8Gipec8Eeeu0xXdbba9frFj0=OqFfea0dXdd9vqai=hGuQ8kuc9pgc9s8qqaq=dirpe0xb9q8qiLsFr0=vr0=vr0dc8meaabaqaciaacaGaaeqabaqabeGadaaakeaacuWGNbWzgaqcamaaBaaaleaacqaIWaamaeqaaaaa@2F2D@ = *g *- *ρ*(*β*), the optimal value of *β *should be determined as

β^=arg maxβρ(β)
 MathType@MTEF@5@5@+=feaafiart1ev1aaatCvAUfKttLearuWrP9MDH5MBPbIqV92AaeXatLxBI9gBaebbnrfifHhDYfgasaacH8akY=wiFfYdH8Gipec8Eeeu0xXdbba9frFj0=OqFfea0dXdd9vqai=hGuQ8kuc9pgc9s8qqaq=dirpe0xb9q8qiLsFr0=vr0=vr0dc8meaabaqaciaacaGaaeqabaqabeGadaaakeaaiiGacuWFYoGygaqcaiabg2da9maaxababaGaeeyyaeMaeeOCaiNaee4zaCMaeeiiaaIaeeyBa0MaeeyyaeMaeeiEaGhaleaacqWFYoGyaeqaaOGae8xWdiNaeiikaGIae8NSdigcbaGae4xkaKcaaa@3F48@

and the method of estimating *g*_0 _is equivalent to finding the optimal *β *value.

The estimation of *g*_0 _can thus be summarized as

### Alg. 2 global-*p *method in *g*_0 _estimation

1. List *r*_β _as the integer such that *p*^(*s*-1) ^≤ *β *∀ *s *= 1,..., *r*_β_, and p(rβ)
 MathType@MTEF@5@5@+=feaafiart1ev1aaatCvAUfKttLearuWrP9MDH5MBPbIqV92AaeXatLxBI9gBaebbnrfifHhDYfgasaacH8akY=wiFfYdH8Gipec8Eeeu0xXdbba9frFj0=OqFfea0dXdd9vqai=hGuQ8kuc9pgc9s8qqaq=dirpe0xb9q8qiLsFr0=vr0=vr0dc8meaabaqaciaacaGaaeqabaqabeGadaaakeaacqWGWbaCdaahaaWcbeqaaiabcIcaOiabdkhaYnaaBaaameaaiiGacqWFYoGyaeqaaSGaeiykaKcaaaaa@3341@ > *β *for a large number of *β*s for 0 <*β *< 1.

2. Find *β *such that *ρ*(*β*) = *r*_*β *_- *β*/(1 - *β*)^2 ^is maximized (see Figure 5). Let this *β *be β*.

3. Let *r*_β* _be the integer such that *p*^(*s*-1) ^≤ β* ∀ *s *= 1,..., *r*_β*_, and p(rβ*)
 MathType@MTEF@5@5@+=feaafiart1ev1aaatCvAUfKttLearuWrP9MDH5MBPbIqV92AaeXatLxBI9gBaebbnrfifHhDYfgasaacH8akY=wiFfYdH8Gipec8Eeeu0xXdbba9frFj0=OqFfea0dXdd9vqai=hGuQ8kuc9pgc9s8qqaq=dirpe0xb9q8qiLsFr0=vr0=vr0dc8meaabaqaciaacaGaaeqabaqabeGadaaakeaacqWGWbaCdaahaaWcbeqaaiabcIcaOiabdkhaYnaaBaaameaaiiGacqWFYoGycqGGQaGkaeqaaSGaeiykaKcaaaaa@341D@ > β*

4. Let g^0
 MathType@MTEF@5@5@+=feaafiart1ev1aaatCvAUfKttLearuWrP9MDH5MBPbIqV92AaeXatLxBI9gBaebbnrfifHhDYfgasaacH8akY=wiFfYdH8Gipec8Eeeu0xXdbba9frFj0=OqFfea0dXdd9vqai=hGuQ8kuc9pgc9s8qqaq=dirpe0xb9q8qiLsFr0=vr0=vr0dc8meaabaqaciaacaGaaeqabaqabeGadaaakeaacuWGNbWzgaqcamaaBaaaleaacqaIWaamaeqaaaaa@2F2D@ = min[*g *- *r*_β* _+ β* /(1 - β*)^2^, *g*].

We called this *global-p *method and denoted it by ℘
 MathType@MTEF@5@5@+=feaafiart1ev1aaatCvAUfKttLearuWrP9MDH5MBPbIqV92AaeXatLxBI9gBaebbnrfifHhDYfgasaacH8akY=wiFfYdH8Gipec8Eeeu0xXdbba9frFj0=OqFfea0dXdd9vqai=hGuQ8kuc9pgc9s8qqaq=dirpe0xb9q8qiLsFr0=vr0=vr0dc8meaabaqaciaacaGaaeqabaqabeGadaaakeaacqGHyeYWaaa@2E34@.

### Alg. 3 Differentially expressed gene selection with given FDR

With *g*_0 _estimated, to control the FDR at *α *level for identifying differentially expressed genes in microarray data analysis with the conservative estimate g^0
 MathType@MTEF@5@5@+=feaafiart1ev1aaatCvAUfKttLearuWrP9MDH5MBPbIqV92AaeXatLxBI9gBaebbnrfifHhDYfgasaacH8akY=wiFfYdH8Gipec8Eeeu0xXdbba9frFj0=OqFfea0dXdd9vqai=hGuQ8kuc9pgc9s8qqaq=dirpe0xb9q8qiLsFr0=vr0=vr0dc8meaabaqaciaacaGaaeqabaqabeGadaaakeaacuWGNbWzgaqcamaaBaaaleaacqaIWaamaeqaaaaa@2F2D@, we simply test each sub-hypothesis based on (1) with the new *α *≡ *α*/g^0
 MathType@MTEF@5@5@+=feaafiart1ev1aaatCvAUfKttLearuWrP9MDH5MBPbIqV92AaeXatLxBI9gBaebbnrfifHhDYfgasaacH8akY=wiFfYdH8Gipec8Eeeu0xXdbba9frFj0=OqFfea0dXdd9vqai=hGuQ8kuc9pgc9s8qqaq=dirpe0xb9q8qiLsFr0=vr0=vr0dc8meaabaqaciaacaGaaeqabaqabeGadaaakeaacuWGNbWzgaqcamaaBaaaleaacqaIWaamaeqaaaaa@2F2D@, i.e., rejects all *H*_(*j*)_, *j *= 1,..., *J*, if *p*_(*j*) _≤ *j *× αg^0
 MathType@MTEF@5@5@+=feaafiart1ev1aaatCvAUfKttLearuWrP9MDH5MBPbIqV92AaeXatLxBI9gBaebbnrfifHhDYfgasaacH8akY=wiFfYdH8Gipec8Eeeu0xXdbba9frFj0=OqFfea0dXdd9vqai=hGuQ8kuc9pgc9s8qqaq=dirpe0xb9q8qiLsFr0=vr0=vr0dc8meaabaqaciaacaGaaeqabaqabeGadaaakeaadaWcaaqaaGGaciab=f7aHbqaaiqbdEgaNzaajaWaaSbaaSqaaiabicdaWaqabaaaaaaa@30E3@ and *p*_(*J*+1) _> (*J *+ 1) × αg^0
 MathType@MTEF@5@5@+=feaafiart1ev1aaatCvAUfKttLearuWrP9MDH5MBPbIqV92AaeXatLxBI9gBaebbnrfifHhDYfgasaacH8akY=wiFfYdH8Gipec8Eeeu0xXdbba9frFj0=OqFfea0dXdd9vqai=hGuQ8kuc9pgc9s8qqaq=dirpe0xb9q8qiLsFr0=vr0=vr0dc8meaabaqaciaacaGaaeqabaqabeGadaaakeaadaWcaaqaaGGaciab=f7aHbqaaiqbdEgaNzaajaWaaSbaaSqaaiabicdaWaqabaaaaaaa@30E3@. We also need to address the choices for the combining function *h*. In this paper, we consider only two commonly used ones. 1) Fisher's sum of logarithm

η=h(p1…,pg)=−2∑j=1glog(pj)
 MathType@MTEF@5@5@+=feaafiart1ev1aaatCvAUfKttLearuWrP9MDH5MBPbIqV92AaeXatLxBI9gBaebbnrfifHhDYfgasaacH8akY=wiFfYdH8Gipec8Eeeu0xXdbba9frFj0=OqFfea0dXdd9vqai=hGuQ8kuc9pgc9s8qqaq=dirpe0xb9q8qiLsFr0=vr0=vr0dc8meaabaqaciaacaGaaeqabaqabeGadaaakeaaiiGacqWF3oaAcqGH9aqpcqWGObaAdaqadaqaaiabdchaWnaaBaaaleaacqaIXaqmaeqaaOGaeSOjGSKaeiilaWIaemiCaa3aaSbaaSqaaiabdEgaNbqabaaakiaawIcacaGLPaaacqGH9aqpcqGHsislcqaIYaGmdaaeWbqaaiabbYgaSjabb+gaVjabbEgaNnaabmaabaGaemiCaa3aaSbaaSqaaiabdQgaQbqabaaakiaawIcacaGLPaaaaSqaaiabdQgaQjabg2da9iabigdaXaqaaiabdEgaNbqdcqGHris5aaaa@4C3D@

and 2) Liptak's sum of inverse standard Gaussian distribution functions

η=h(p1,…,pg)=∑j=1gΦ−1(1−pj).
 MathType@MTEF@5@5@+=feaafiart1ev1aaatCvAUfKttLearuWrP9MDH5MBPbIqV92AaeXatLxBI9gBaebbnrfifHhDYfgasaacH8akY=wiFfYdH8Gipec8Eeeu0xXdbba9frFj0=OqFfea0dXdd9vqai=hGuQ8kuc9pgc9s8qqaq=dirpe0xb9q8qiLsFr0=vr0=vr0dc8meaabaqaciaacaGaaeqabaqabeGadaaakeaaiiGacqWF3oaAcqGH9aqpcqWGObaAdaqadaqaaiabdchaWnaaBaaaleaacqaIXaqmaeqaaOGaeiilaWIaeSOjGSKaeiilaWIaemiCaa3aaSbaaSqaaiabdEgaNbqabaaakiaawIcacaGLPaaacqGH9aqpdaaeWbqaaiabfA6agnaaCaaaleqabaGaeyOeI0IaeGymaedaaOWaaeWaaeaacqaIXaqmcqGHsislcqWGWbaCdaWgaaWcbaGaemOAaOgabeaaaOGaayjkaiaawMcaaaWcbaGaemOAaOMaeyypa0JaeGymaedabaGaem4zaCganiabggHiLdGccqGGUaGlaaa@4D7E@

More discussion on the choices of combining functions can be found in Birnbaum [15] and Folks [16]. The computer program for the proposed method, global-*p*, has been implemented in R script and is publicly available [17].

## Results

### Simulation

Two simulation studies were conducted. First, when the number of hypotheses are small and second, when the total number of hypotheses is extremely large. For small number of hypotheses, we used the following configurations: numbers of hypotheses are 16, 32, 64, and 128 with sample size *n *= 10 and the proportions of *g*_0_/*g *being 0, 0.25, 0.5 and 0.75. The true expressions under the alternative hypotheses are assumed to be variance 1 Gaussian random variables with non-zero mean values *μ*_*j *_= *d*_*j*_. In one set of experiments, all *d*_*j*_s are set as 0.2 or 0.4; in the other set the *d*_*j*_s are divided into 4 equal size blocks with values 0.2, 0.4, 0.6 or 0.8 in each block. The number of permutations is *B *= 1,000 and the number of simulations is 20,000. The FDR is set at *α *= 0.05. Four procedures are used for testing: BH, aBH, our global-p test one with Fisher's combining function, and one with Liptak's combining function. For the purpose of comparison, we also plug in the true value of *g*_0 _into the aBH method. This is the ideal situation to reach the highest power.

**Figure 1:**
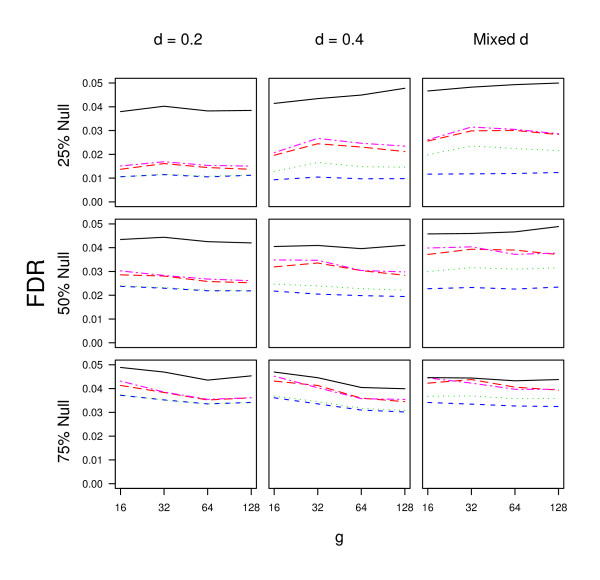
A simulation study to estimate FDR (in y-axis) for various numbers of hypotheses Tested (x-axis), proportion of true null hypotheses (left labels), and various configurations of true alternative hypotheses (top labels). All the examined methods, BH procedure (- - -); aBH procedure (......); Fisher's combining function (-- --); Liptak's combining function (-- . --); proportion of null known (----), satisfied the required FDR ≤ 0.05.

**Figure 2:**
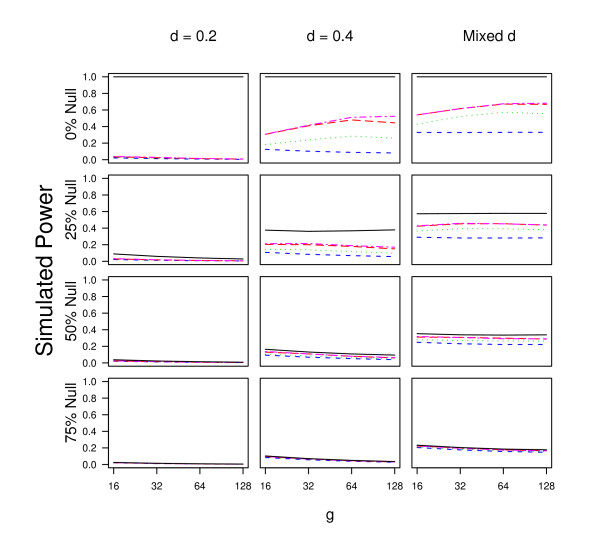
The same simulation study with estimated average powers, using the same legends as Figure 1. The powers of the new method are clearly higher than the existing two, especially when the null proportion is small.

Table 1 lists the simulated expected FDR when all null hypotheses are true. Figures 1 and 2 show the estimated FDR and power under various gene expression compositions.

Based on this simulation results, all the four methods have their expected FDR below the nominal FDR *α *= 0.05. Both combining functions in our proposed method have a higher power than the aBH procedure in most situations, while there is little difference between them. The improvement over the aBH methods is substantial when the proportion of the true null hypotheses is small.

Since most microarray data consist of tens of thousands of genes simultaneously and many of their expressions are correlated, our second simulation tried to reflect these facts. Storey and Tibshirani [13] proposed a "clumpy" dependence model in which genes are partitioned into blocks. The gene expressions are assumed independent between blocks but dependent within the same block. Following the clumpy model, 10 test and 10 control samples were generated from normal random variables with mean 0 and standard deviation 1 where each sample contained 10,000 genes. For test samples, genes with expression difference were added by 3 to represent the expression differences. The number of differentially expressed genes *g*_0 _were 100, 2,000, 5,000, 8,000 which corresponded to the proportion of nulls *π*_0 _at 0.99, 0.8, 0.5, 0.2. To simulate intra-block dependency, we generated one vector of normal random variables with mean 0 and standard deviation 0.2 in each block of size 50 and add it to every gene in that block. This process creates correlations between genes. Since the true expression differences for non-null genes are moderate large in this simulation, we expect that any good method should accurately estimate *π*_0_.

In a recent article, Broberg [18] did a comparative review of various newly developed methods for estimating *π*_0_. To make a comparison, we presented the means and standard errors of estimates of *π*_0 _for all the methods describe in Broberg's paper in addition to our proposed method (℘
 MathType@MTEF@5@5@+=feaafiart1ev1aaatCvAUfKttLearuWrP9MDH5MBPbIqV92AaeXatLxBI9gBaebbnrfifHhDYfgasaacH8akY=wiFfYdH8Gipec8Eeeu0xXdbba9frFj0=OqFfea0dXdd9vqai=hGuQ8kuc9pgc9s8qqaq=dirpe0xb9q8qiLsFr0=vr0=vr0dc8meaabaqaciaacaGaaeqabaqabeGadaaakeaacqGHyeYWaaa@2E34@) over 1000 simulations. The details of *π*_0_estimation methods (BUM, SPLOSH, QVALUE, Boostrap LSE, SEP, LSL, mgf, PRE) can be found in Broberg's paper and the programs for these methods can be found in the add-on packages of the freely available R software [19]. The simulation results are shown in Table 2.

As expected, all of them performed very well for large *π*_0_. If both the mean (bias) and standard error (stability) over the whole range of *π*_0 _are considered, LSL and Global-p stand out and LSL is better in stability. However, as we will see from the next study, LSL seems to be over-conservative in real data analysis. The means of both combining functions of our proposed method performed well but Liptak's function has a higher standard errors. Therefore, Fisher's function will be used for the real data analysis. During the simulation, we also noticed that current implementation of SPLOSH, SEP, mgf, and PRE methods in R programs failed to work when the number of hypotheses was small.

### Real data analysis

We use a publically available experimental data set from the Stanford microarray database [20] first to compare the differences between BH, aBH and our method with Fisher's combining function. The purpose of this experiment was to identify genes that have different expressions between prostate tumor tissue and matched normal tissue. It consists of a total of 82 microarrays: 41 arrays were from primary prostate tumors and the other half were from matched normal prostate tissues. Each array contains 32,152 different human genes. We chose this data set because of the large number of replicates. With this amount of replicates, we had a better idea of the ground truth.

To compare performances of various methods, we split the whole data set into two groups: a test data set and a confirmation data set. Eleven pairs of tumor and normal arrays were chosen for the test data set and the remaining arrays were used for the confirmation. The number of eleven test pairs were taken from a systematic sampling to avoid bias. Patients 1, 5, 9,..., 41 in the original order from the database were chosen as the test set. Expression information is missing if it is labelled as failed, contaminated, or flagged. Genes were removed from our study if more than half of their expressions were missing in the original data set. A total of 24,865 genes was used to identify differences in expressions using BH, aBH and our proposed procedures at 0.01 expected FDR level. Since the experiment was designed with paired tumor-normal arrays, the paired *t*-test was used to derive the *p*-value of each gene in the test data set. The BH procedure identified 1,254 genes, the aBH procedure identified 1,523 genes, and our proposed method identified 2,119 genes. Our test is apparently more powerful if it can maintain the required FDR 0.01. Since we did not have the biological information, another approach was to estimate FDR based on our rejection set. Specifically, suppose the rejection set contains the *p*-values that are less than *ξ *and the estimated proportion of null hypotheses is π^0
 MathType@MTEF@5@5@+=feaafiart1ev1aaatCvAUfKttLearuWrP9MDH5MBPbIqV92AaeXatLxBI9gBaebbnrfifHhDYfgasaacH8akY=wiFfYdH8Gipec8Eeeu0xXdbba9frFj0=OqFfea0dXdd9vqai=hGuQ8kuc9pgc9s8qqaq=dirpe0xb9q8qiLsFr0=vr0=vr0dc8meaabaqaciaacaGaaeqabaqabeGadaaakeaaiiGacuWFapaCgaqcamaaBaaaleaaiiaacqGFWaamaeqaaaaa@2F9A@. An intuitive estimate for FDR is [21,22]

FDR^(ξ)=π^0ξPr^[P≤ξ],     (4)
 MathType@MTEF@5@5@+=feaafiart1ev1aaatCvAUfKttLearuWrP9MDH5MBPbIqV92AaeXatLxBI9gBaebbnrfifHhDYfgasaacH8akY=wiFfYdH8Gipec8Eeeu0xXdbba9frFj0=OqFfea0dXdd9vqai=hGuQ8kuc9pgc9s8qqaq=dirpe0xb9q8qiLsFr0=vr0=vr0dc8meaabaqaciaacaGaaeqabaqabeGadaaakeaadaqiaaqaaiabbAeagjabbseaejabbkfasbGaayPadaGaeiikaGccciGae8NVdGNaeiykaKIaeyypa0ZaaSaaaeaacuWFapaCgaqcamaaBaaaleaacqaIWaamaeqaaOGae8NVdGhabaWaaecaaeaatCvAUfeBSjuyZL2yd9gzLbvyNv2CaeHbwvMCKfMBHbaceiGaa4huaiaa+jhaaiaawkWaaiabcUfaBjabdcfaqjabgsMiJkab=57a4jabc2faDbaacqGGSaalcaWLjaGaaCzcamaabmaabaGaeGinaqdacaGLOaGaayzkaaaaaa@52C7@

where Pr^[P≤ξ]=∑j=1gI(pi≤ξ)/g
 MathType@MTEF@5@5@+=feaafiart1ev1aaatCvAUfeBSjuyZL2yd9gzLbvyNv2CaerbwvMCKfMBHbqedmvETj2BSbqee0evGueE0jxyaibaieYdOi=BH8vipeYdI8qiW7rqqrFfpeea0xe9Lq=Jc9vqaqpepm0xbbG8FasPYRqj0=yi0lXdbba9pGe9qqFf0dXdHuk9fr=xfr=xfrpiWZqaaeaabiGaaiaacaqabeaabeqacmaaaOqaamaaHaaabaacbiGaa8huaiaa=jhaaiaawkWaaiaacUfacaWGqbGaeyizImkcciGae4NVdGNaaiyxaiabg2da9maaqadabaGaamysaiaacIcacaWGWbWaaSbaaSqaaiaadMgaaeqaaaqaaiaadQgacqGH9aqpcaaIXaaabaGaam4zaaqdcqGHris5aOGaeyizImQae4NVdGNaaiykaiaac+cacaWGNbaaaa@4850@. Based on the confirm data set, our proposed method reported an estimator of *π*_0_, π^0
 MathType@MTEF@5@5@+=feaafiart1ev1aaatCvAUfKttLearuWrP9MDH5MBPbIqV92AaeXatLxBI9gBaebbnrfifHhDYfgasaacH8akY=wiFfYdH8Gipec8Eeeu0xXdbba9frFj0=OqFfea0dXdd9vqai=hGuQ8kuc9pgc9s8qqaq=dirpe0xb9q8qiLsFr0=vr0=vr0dc8meaabaqaciaacaGaaeqabaqabeGadaaakeaaiiGacuWFapaCgaqcamaaBaaaleaaiiaacqGFWaamaeqaaaaa@2F9A@ = 0.5326. Using equation (4), the estimated FDRs for BH, aBH and our method were 0.0054, 0.0067, 0.0099, respectively.

If we further reject more sub-hypotheses beyond the number provided by our method, the FDR will exceed the assigned level. For example, if we reject the next 500 null sub-hypotheses that have the smallest *p*-values among those not previously rejected, the estimated FDR is 0.0137 which is larger than the pre-assigned 0.01 level.

**Figure 3:**
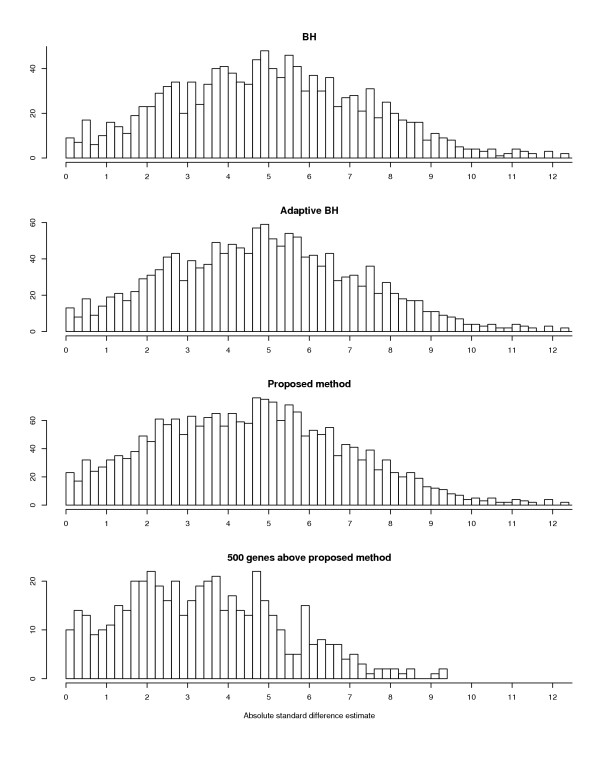
The histograms of the absolute standard difference estimates (*x*-axis) in the confirmation data set for the selected genes from the three procedures based on the test data set for the prostate tumor data. The top is the selected gene histogram by the BH procedure; the second is that by the aBH procdure; and the third is the one by our method. The similarity of the three histograms means that the additional 596 genes picked by our method should maintain the same FDR (0.01) as the other two. The bottom panel contains the next 500 genes beyond our proposed method. The left shift of this histogram means that the additional genes may contain too many false discoveries.

Moreover, we calculated the standard difference (paired *t*-statistic) of expressions using the confirmation data set. The confirmation data set contains 30 pairs of arrays so that the statistic is a good estimate for the unknown standard difference. Figure 3 shows histograms of absolute standard differences based on genes identified by the BH procedure, by the aBH procedure, by our method, and the extra 500 genes beyond our method. From the histograms, several hundred differentially expressed genes that were not identified by BH or aBH in the test arrays were identified by our method and most of them have standard expression differences greater than 2. However, the next group of 500 genes beyond our method may contain too many false discoveries to make the FDR acceptable.

Next, we use this data set to compare different estimates of proportion of null genes, *π*_0_. The whole data set was partitioned into four sub-data sets. We labelled the arrays from 1 to 41 based on the original order in their database. Sub-data set 1 contained 11 arrays with labels 1, 5, 9,..., 41; sub-data set 2 contains 10 arrays with labels 2,6,..., 38; sub-data set 3 contains 10 arrays with labels 3,7,..., 39; sub-data set 4 contained the remaining arrays. Although we did not know the true value of *π*_0 _in real data analysis, we used the four sub-data sets to compare the variation of different estimation methods. In addition, we used the whole data set to check if the estimates are reliable. For global-*p *method, we also plot the function of *ρ*(*β*) in Figure 5 using the whole data set. The maximum of *ρ*(*β*) occurs at *β *= 0.91.

**Figure 4:**
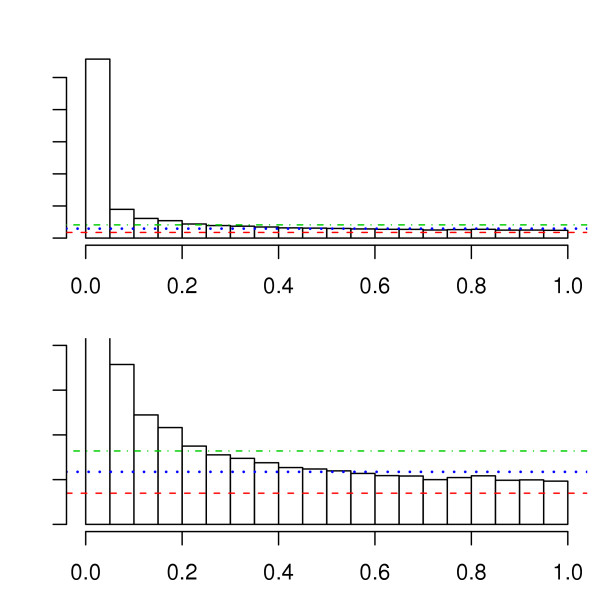
The histogram of *p*-values in a real data analysis. About half of the large *p*-values to the right look uniformly distributed.

**Figure 5:**
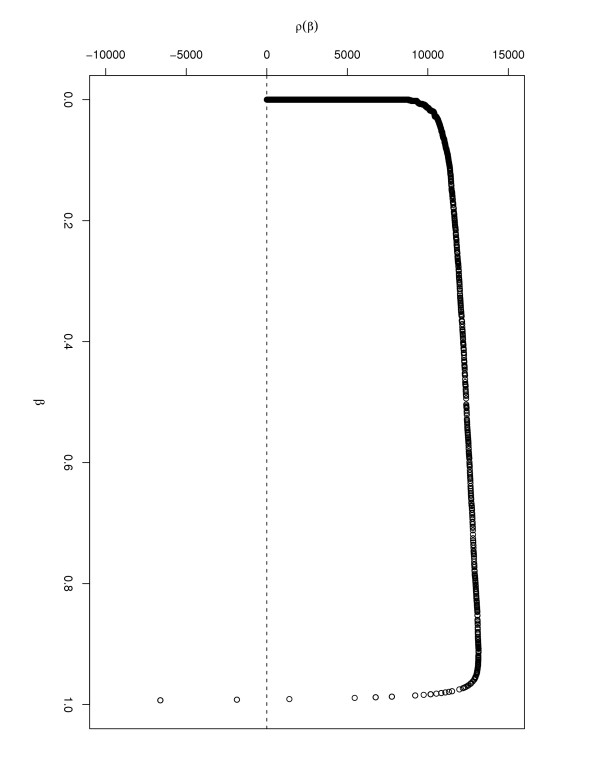
The plot of function *ρ*(*β*) in a real data analysis to determine the arg max *ρ*(*β*) for the tumor data.

The estimates π^0
 MathType@MTEF@5@5@+=feaafiart1ev1aaatCvAUfKttLearuWrP9MDH5MBPbIqV92AaeXatLxBI9gBaebbnrfifHhDYfgasaacH8akY=wiFfYdH8Gipec8Eeeu0xXdbba9frFj0=OqFfea0dXdd9vqai=hGuQ8kuc9pgc9s8qqaq=dirpe0xb9q8qiLsFr0=vr0=vr0dc8meaabaqaciaacaGaaeqabaqabeGadaaakeaaiiGacuWFapaCgaqcamaaBaaaleaaiiaacqGFWaamaeqaaaaa@2F9A@ were listed in Table 3. All π^0
 MathType@MTEF@5@5@+=feaafiart1ev1aaatCvAUfKttLearuWrP9MDH5MBPbIqV92AaeXatLxBI9gBaebbnrfifHhDYfgasaacH8akY=wiFfYdH8Gipec8Eeeu0xXdbba9frFj0=OqFfea0dXdd9vqai=hGuQ8kuc9pgc9s8qqaq=dirpe0xb9q8qiLsFr0=vr0=vr0dc8meaabaqaciaacaGaaeqabaqabeGadaaakeaaiiGacuWFapaCgaqcamaaBaaaleaaiiaacqGFWaamaeqaaaaa@2F9A@ estimates in the subsets were larger than the ones in the whole data set. This is expected because genes with small expressional differences are more likely to be classified as null genes in a small sample. From the *π*_0 _estimates from the whole data set, we may say the LSL gave a large *π*_0 _value, PRE gave a small value and all the others gave similar estimates. We used LSL, PRE and Global-p to draw the *p*-value density histogram using the whole data set in Figure 4. The upper panel is the overall view and the lower panel is the closer view. The green dash-dot line is the height using LSL estimate; the red dash line PRE estimate; the blue dot line global-*p *estimate. The LSL method, which is also shown in Broberg's study, is too conservative producing the largest value. The PRE method underestimated *π*_0_. If we used π^0
 MathType@MTEF@5@5@+=feaafiart1ev1aaatCvAUfKttLearuWrP9MDH5MBPbIqV92AaeXatLxBI9gBaebbnrfifHhDYfgasaacH8akY=wiFfYdH8Gipec8Eeeu0xXdbba9frFj0=OqFfea0dXdd9vqai=hGuQ8kuc9pgc9s8qqaq=dirpe0xb9q8qiLsFr0=vr0=vr0dc8meaabaqaciaacaGaaeqabaqabeGadaaakeaaiiGacuWFapaCgaqcamaaBaaaleaaiiaacqGFWaamaeqaaaaa@2F9A@ by PRE method to improve FDR procedure, it is likely to have a higher FDR than the nominal level. We think our *π*_0 _estimate is reliable because it is close but higher than the height of observed *p*-values in the region of (0.6, 1) if we assume genes with expressional differences have rare chance to have large observed *p*-values.

## Conclusion

Benjamini and Hochberg's FDR procedure [2] opens an useful approach for multiple comparisons in microarray analysis. Due to the complexity in microarray experiments, it seems unlikely that one method can dominate all the other methods in all the cases. In this paper, we have demonstrated a new method that is intuitive appearing because:

1. It uses permutation test which can take care the complex correlation structures in gene expressions.

2. Its global test based on sequentially eliminated significant genes should provide a good stopping rule because all the remaining genes are always considered together.

With the support of simulation and real data studies, the new method should be a viable alternative to find the differentially expressed genes in microarray experiments.

## Algorithm

We illustrate the algorithm for calculating pseudo-global *p*-values based on marginal *p*-values, *p*_1_,..., pg0
 MathType@MTEF@5@5@+=feaafiart1ev1aaatCvAUfKttLearuWrP9MDH5MBPbIqV92AaeXatLxBI9gBaebbnrfifHhDYfgasaacH8akY=wiFfYdH8Gipec8Eeeu0xXdbba9frFj0=OqFfea0dXdd9vqai=hGuQ8kuc9pgc9s8qqaq=dirpe0xb9q8qiLsFr0=vr0=vr0dc8meaabaqaciaacaGaaeqabaqabeGadaaakeaacqWGWbaCdaWgaaWcbaGaem4zaC2aaSbaaWqaaiabicdaWaqabaaaleqaaaaa@30BE@. Please note that the procedure described here is the same regardless of *g*_0_. We consider *h*(*p*_1_, ..., pg0
 MathType@MTEF@5@5@+=feaafiart1ev1aaatCvAUfKttLearuWrP9MDH5MBPbIqV92AaeXatLxBI9gBaebbnrfifHhDYfgasaacH8akY=wiFfYdH8Gipec8Eeeu0xXdbba9frFj0=OqFfea0dXdd9vqai=hGuQ8kuc9pgc9s8qqaq=dirpe0xb9q8qiLsFr0=vr0=vr0dc8meaabaqaciaacaGaaeqabaqabeGadaaakeaacqWGWbaCdaWgaaWcbaGaem4zaC2aaSbaaWqaaiabicdaWaqabaaaleqaaaaa@30BE@) in the summation form:

η=h(p1,…,pg0)=∑i=1g0ℏ(pi),     (5)
 MathType@MTEF@5@5@+=feaafiart1ev1aaatCvAUfKttLearuWrP9MDH5MBPbIqV92AaeXatLxBI9gBaebbnrfifHhDYfgasaacH8akY=wiFfYdH8Gipec8Eeeu0xXdbba9frFj0=OqFfea0dXdd9vqai=hGuQ8kuc9pgc9s8qqaq=dirpe0xb9q8qiLsFr0=vr0=vr0dc8meaabaqaciaacaGaaeqabaqabeGadaaakeaaiiGacqWF3oaAcqGH9aqpcqWGObaAcqGGOaakcqWGWbaCdaWgaaWcbaGaeGymaedabeaakiabcYcaSiablAciljabcYcaSiabdchaWnaaBaaaleaacqWGNbWzdaWgaaadbaGaeGimaadabeaaaSqabaGccqGGPaqkcqGH9aqpdaaeWbqaaiabl+qiObWcbaGaemyAaKMaeyypa0JaeGymaedabaGaem4zaC2aaSbaaWqaaiabicdaWaqabaaaniabggHiLdGccqGGOaakcqWGWbaCdaWgaaWcbaGaemyAaKgabeaakiabcMcaPiabcYcaSiaaxMaacaWLjaWaaeWaaeaacqaI1aqnaiaawIcacaGLPaaaaaa@4F8C@

where *ħ *is a differentiable decreasing function in which ℏ'(p)=dℏ(t)dt|t=p
 MathType@MTEF@5@5@+=feaafiart1ev1aaatCvAUfKttLearuWrP9MDH5MBPbIqV92AaeXatLxBI9gBaebbnrfifHhDYfgasaacH8akY=wiFfYdH8Gipec8Eeeu0dXdbba9frFj0=OqFfea0dXdd9vqai=hGuQ8kuc9pgc9s8qqaq=dirpe0xb9q8qiLsFr0=vr0=vr0dc8meaabaqaciaacaGaaeqabaqabeGadaaakeaacqWIpecAcqGGNaWjcqGGOaakcqWGWbaCcqGGPaqkcqGH9aqpdaWcaaqaaiabdsgaKjabl+qiOjabcIcaOiabdsha0jabcMcaPaqaaiabdsgaKjabdsha0baadaabbaqaauaabeqaceaaaeaaaeaacqWG0baDcqGH9aqpcqWGWbaCaaaacaGLhWoaaaa@40B7@ exists and *ħ*(*p*) → ∞ as *p *→ 0. Note that equation (5) totally meets the requirement that *h *should be a differentiable real-valued decreasing function. The realization of *ħ *can be, for instance, *ħ*(*p*) = -2log(*p*) if we use Fisher's sum of logarithm or *ħ*(*p*) = Φ^-1 ^(1 - *p*) if we use Liptak's sum of inverse standard Gaussian distribution function. Recall that *p*_*j *_is the *p*-value obtained from *j*th row vector (*Y*_1*j*_,..., *Y*_*nj*_) and the ordered marginal *p*-values are *p*_(1) _≤ *p*_(2)_≤ .... ≤ p(g0)
 MathType@MTEF@5@5@+=feaafiart1ev1aaatCvAUfKttLearuWrP9MDH5MBPbIqV92AaeXatLxBI9gBaebbnrfifHhDYfgasaacH8akY=wiFfYdH8Gipec8Eeeu0xXdbba9frFj0=OqFfea0dXdd9vqai=hGuQ8kuc9pgc9s8qqaq=dirpe0xb9q8qiLsFr0=vr0=vr0dc8meaabaqaciaacaGaaeqabaqabeGadaaakeaacqWGWbaCdaWgaaWcbaGaeiikaGIaem4zaC2aaSbaaWqaaiabicdaWaqabaWccqGGPaqkaeqaaaaa@3270@. The *p*-values for individual genes are denoted by subscripts while the pseudo-global *p*-values in (2) in are denoted by superscripts. Parentheses are used to reveal the order property.

In one-sample testing problem, we permute data *B *times, where each time we assign *w*_*i *_= ± 1 with equal probability to (*w*_1_***Y***_1_,..., *w*_*n*_***Y***_*n*_). For each permuted data, we use *p*_*b*(*j*) _to be the *p*-value from the *b*th permuted data for the gene that produced *p*_(*j*)_, *j *= 1,..., *g*_0_, *b *= 1,..., *B*.

We summarize *p*_(*i*) _and *p*_*b*(*i*) _using the following table.

p(1)p1(1)…pb(1)…pB(1)p(2)p1(2)…pb(2)…pB(2)⋮⋮⋮⋮⋮⋮p(g0)p1(g0)…pb(g0)…pB(g0)     (6)
 MathType@MTEF@5@5@+=feaafiart1ev1aaatCvAUfKttLearuWrP9MDH5MBPbIqV92AaeXatLxBI9gBaebbnrfifHhDYfgasaacH8akY=wiFfYdH8Gipec8Eeeu0xXdbba9frFj0=OqFfea0dXdd9vqai=hGuQ8kuc9pgc9s8qqaq=dirpe0xb9q8qiLsFr0=vr0=vr0dc8meaabaqaciaacaGaaeqabaqabeGadaaakeaafaqabeabgaaaaaqaaiabdchaWnaaBaaaleaacqGGOaakcqaIXaqmcqGGPaqkaeqaaaGcbaGaemiCaa3aaSbaaSqaaiabigdaXiabcIcaOiabigdaXiabcMcaPaqabaaakeaacqWIMaYsaeaacqWGWbaCdaWgaaWcbaGaemOyaiMaeiikaGIaeGymaeJaeiykaKcabeaaaOqaaiablAcilbqaaiabdchaWnaaBaaaleaacqWGcbGqcqGGOaakcqaIXaqmcqGGPaqkaeqaaaGcbaGaemiCaa3aaSbaaSqaaiabcIcaOiabikdaYiabcMcaPaqabaaakeaacqWGWbaCdaWgaaWcbaGaeGymaeJaeiikaGIaeGOmaiJaeiykaKcabeaaaOqaaiablAcilbqaaiabdchaWnaaBaaaleaacqWGIbGycqGGOaakcqaIYaGmcqGGPaqkaeqaaaGcbaGaeSOjGSeabaGaemiCaa3aaSbaaSqaaiabdkeacjabcIcaOiabikdaYiabcMcaPaqabaaakeaacqWIUlstaeaacqWIUlstaeaacqWIUlstaeaacqWIUlstaeaacqWIUlstaeaacqWIUlstaeaacqWGWbaCdaWgaaWcbaGaeiikaGIaem4zaC2aaSbaaWqaaiabicdaWaqabaWccqGGPaqkaeqaaaGcbaGaemiCaa3aaSbaaSqaaiabigdaXiabcIcaOiabdEgaNnaaBaaameaacqaIWaamaeqaaSGaeiykaKcabeaaaOqaaiablAcilbqaaiabdchaWnaaBaaaleaacqWGIbGycqGGOaakcqWGNbWzdaWgaaadbaGaeGimaadabeaaliabcMcaPaqabaaakeaacqWIMaYsaeaacqWGWbaCdaWgaaWcbaGaemOqaiKaeiikaGIaem4zaC2aaSbaaWqaaiabicdaWaqabaWccqGGPaqkaeqaaaaakiaaxMaacaWLjaWaaeWaaeaacqaI2aGnaiaawIcacaGLPaaaaaa@8623@

Note that the first column is *p*-values of genes from the original data while the remaining columns are from the permuted data.

To simplify the notation, let *ħ*(*p*_(*i*)_) = *ħ*_(*i*)_, *ħ*(*p*_*b*(*i*)_) = *ħ*_*b*(*i*) _and *η *is just the sum of *ħ*_(·)_. Therefore, we can transform the table (6) into the following table with the addition of the last row where its value *η *is the sum of the *ħ*_(·) _in its corresponding column.

ℏ(1)ℏ1(1)…ℏb(1)…ℏB(1)ℏ(2)ℏ1(2)…ℏb(2)…ℏB(2)⋮⋮⋮⋮⋮⋮ℏ(g0)ℏ1(g0)…ℏb(g0)…ℏB(g0)⇓⇓…⇓…⇓η(0)η1(0)…ηb(0)…ηB(0)     (7)
 MathType@MTEF@5@5@+=feaafiart1ev1aaatCvAUfKttLearuWrP9MDH5MBPbIqV92AaeXatLxBI9gBaebbnrfifHhDYfgasaacH8akY=wiFfYdH8Gipec8Eeeu0xXdbba9frFj0=OqFfea0dXdd9vqai=hGuQ8kuc9pgc9s8qqaq=dirpe0xb9q8qiLsFr0=vr0=vr0dc8meaabaqaciaacaGaaeqabaqabeGadaaakeaafaqabeGbgaaaaaqaaiabl+qiOnaaBaaaleaacqGGOaakcqaIXaqmcqGGPaqkaeqaaaGcbaGaeS4dHG2aaSbaaSqaaiabigdaXiabcIcaOiabigdaXiabcMcaPaqabaaakeaacqWIMaYsaeaacqWIpecAdaWgaaWcbaGaemOyaiMaeiikaGIaeGymaeJaeiykaKcabeaaaOqaaiablAcilbqaaiabl+qiOnaaBaaaleaacqWGcbGqcqGGOaakcqaIXaqmcqGGPaqkaeqaaaGcbaGaeS4dHG2aaSbaaSqaaiabcIcaOiabikdaYiabcMcaPaqabaaakeaacqWIpecAdaWgaaWcbaGaeGymaeJaeiikaGIaeGOmaiJaeiykaKcabeaaaOqaaiablAcilbqaaiabl+qiOnaaBaaaleaacqWGIbGycqGGOaakcqaIYaGmcqGGPaqkaeqaaaGcbaGaeSOjGSeabaGaeS4dHG2aaSbaaSqaaiabdkeacjabcIcaOiabikdaYiabcMcaPaqabaaakeaacqWIUlstaeaacqWIUlstaeaacqWIUlstaeaacqWIUlstaeaacqWIUlstaeaacqWIUlstaeaacqWIpecAdaWgaaWcbaGaeiikaGIaem4zaC2aaSbaaWqaaiabicdaWaqabaWccqGGPaqkaeqaaaGcbaGaeS4dHG2aaSbaaSqaaiabigdaXiabcIcaOiabdEgaNnaaBaaameaacqaIWaamaeqaaSGaeiykaKcabeaaaOqaaiablAcilbqaaiabl+qiOnaaBaaaleaacqWGIbGycqGGOaakcqWGNbWzdaWgaaadbaGaeGimaadabeaaliabcMcaPaqabaaakeaacqWIMaYsaeaacqWIpecAdaWgaaWcbaGaemOqaiKaeiikaGIaem4zaC2aaSbaaWqaaiabicdaWaqabaWccqGGPaqkaeqaaaGcbaGaey40H8nabaGaey40H8nabaGaeSOjGSeabaGaey40H8nabaGaeSOjGSeabaGaey40H8nabaacciGae83TdG2aaWbaaSqabeaacqGGOaakcqaIWaamcqGGPaqkaaaakeaacqWF3oaAdaqhaaWcbaGaeGymaedabaGaeiikaGIaeGimaaJaeiykaKcaaaGcbaGaeSOjGSeabaGae83TdG2aa0baaSqaaiabdkgaIbqaaiabcIcaOiabicdaWiabcMcaPaaaaOqaaiablAcilbqaaiab=D7aOnaaDaaaleaacqWGcbGqaeaacqGGOaakcqaIWaamcqGGPaqkaaaaaOGaaCzcaiaaxMaadaqadaqaaiabiEda3aGaayjkaiaawMcaaaaa@A685@

Once *η*^(0) ^and *η*^(1)^,..., *η*^(*B*) ^are available, the global *p*-value, by definition, is

p(0)=∑b=1BI(ηb(0)≥η(0))B.
 MathType@MTEF@5@5@+=feaafiart1ev1aaatCvAUfKttLearuWrP9MDH5MBPbIqV92AaeXatLxBI9gBaebbnrfifHhDYfgasaacH8akY=wiFfYdH8Gipec8Eeeu0xXdbba9frFj0=OqFfea0dXdd9vqai=hGuQ8kuc9pgc9s8qqaq=dirpe0xb9q8qiLsFr0=vr0=vr0dc8meaabaqaciaacaGaaeqabaqabeGadaaakeaacqWGWbaCdaahaaWcbeqaaiabcIcaOiabicdaWiabcMcaPaaakiabg2da9maalaaabaWaaabmaeaacqWGjbqscqGGOaakiiGacqWF3oaAdaqhaaWcbaGaemOyaigabaGaeiikaGIaeGimaaJaeiykaKcaaaqaaiabdkgaIjabg2da9iabigdaXaqaaiabdkeacbqdcqGHris5aOGaeyyzImRae83TdG2aaWbaaSqabeaacqGGOaakcqaIWaamcqGGPaqkaaGccqGGPaqkaeaacqWGcbGqaaGaeiOla4caaa@4922@

The same process is directly used to calculate the pseudo-global *p*-values after removing the genes which have the smallest *p*-values. Actually, we can use the available data illustrated in table (8). By induction, suppose *p*_(1)_,..., *p*_(*s*) _have been removed, the *ħ *values of the raw data and their reference sets are

ℏ(s+1)ℏ1(s+1)…ℏb(s+1)…ℏB(s+1)ℏ(s+2)ℏ1(s+2)…ℏb(s+2)…ℏB(s+2)⋮⋮⋮⋮⋮⋮ℏ(g0)ℏ1(g0)…ℏb(g0)…ℏB(g0)⇓⇓…⇓…⇓η(s)η1(s)…ηb(s)…ηB(s)     (8)
 MathType@MTEF@5@5@+=feaafiart1ev1aaatCvAUfKttLearuWrP9MDH5MBPbIqV92AaeXatLxBI9gBaebbnrfifHhDYfgasaacH8akY=wiFfYdH8Gipec8Eeeu0xXdbba9frFj0=OqFfea0dXdd9vqai=hGuQ8kuc9pgc9s8qqaq=dirpe0xb9q8qiLsFr0=vr0=vr0dc8meaabaqaciaacaGaaeqabaqabeGadaaakeaafaqabeGbgaaaaaqaaiabl+qiOnaaBaaaleaacqGGOaakcqWGZbWCcqGHRaWkcqaIXaqmcqGGPaqkaeqaaaGcbaGaeS4dHG2aaSbaaSqaaiabigdaXiabcIcaOiabdohaZjabgUcaRiabigdaXiabcMcaPaqabaaakeaacqWIMaYsaeaacqWIpecAdaWgaaWcbaGaemOyaiMaeiikaGIaem4CamNaey4kaSIaeGymaeJaeiykaKcabeaaaOqaaiablAcilbqaaiabl+qiOnaaBaaaleaacqWGcbGqcqGGOaakcqWGZbWCcqGHRaWkcqaIXaqmcqGGPaqkaeqaaaGcbaGaeS4dHG2aaSbaaSqaaiabcIcaOiabdohaZjabgUcaRiabikdaYiabcMcaPaqabaaakeaacqWIpecAdaWgaaWcbaGaeGymaeJaeiikaGIaem4CamNaey4kaSIaeGOmaiJaeiykaKcabeaaaOqaaiablAcilbqaaiabl+qiOnaaBaaaleaacqWGIbGycqGGOaakcqWGZbWCcqGHRaWkcqaIYaGmcqGGPaqkaeqaaaGcbaGaeSOjGSeabaGaeS4dHG2aaSbaaSqaaiabdkeacjabcIcaOiabdohaZjabgUcaRiabikdaYiabcMcaPaqabaaakeaacqWIUlstaeaacqWIUlstaeaacqWIUlstaeaacqWIUlstaeaacqWIUlstaeaacqWIUlstaeaacqWIpecAdaWgaaWcbaGaeiikaGIaem4zaC2aaSbaaWqaaiabicdaWaqabaWccqGGPaqkaeqaaaGcbaGaeS4dHG2aaSbaaSqaaiabigdaXiabcIcaOiabdEgaNnaaBaaameaacqaIWaamaeqaaSGaeiykaKcabeaaaOqaaiablAcilbqaaiabl+qiOnaaBaaaleaacqWGIbGycqGGOaakcqWGNbWzdaWgaaadbaGaeGimaadabeaaliabcMcaPaqabaaakeaacqWIMaYsaeaacqWIpecAdaWgaaWcbaGaemOqaiKaeiikaGIaem4zaC2aaSbaaWqaaiabicdaWaqabaWccqGGPaqkaeqaaaGcbaGaey40H8nabaGaey40H8nabaGaeSOjGSeabaGaey40H8nabaGaeSOjGSeabaGaey40H8nabaacciGae83TdG2aaWbaaSqabeaacqGGOaakcqWGZbWCcqGGPaqkaaaakeaacqWF3oaAdaqhaaWcbaGaeGymaedabaGaeiikaGIaem4CamNaeiykaKcaaaGcbaGaeSOjGSeabaGae83TdG2aa0baaSqaaiabdkgaIbqaaiabcIcaOiabdohaZjabcMcaPaaaaOqaaiablAcilbqaaiab=D7aOnaaDaaaleaacqWGcbGqaeaacqGGOaakcqWGZbWCcqGGPaqkaaaaaOGaaCzcaiaaxMaadaqadaqaaiabiIda4aGaayjkaiaawMcaaaaa@BB13@

and the pseudo-global *p*-value after removing the *s *genes is

p(s)=∑b=1BI(ηb(s)≥η(s))B.
 MathType@MTEF@5@5@+=feaafiart1ev1aaatCvAUfKttLearuWrP9MDH5MBPbIqV92AaeXatLxBI9gBaebbnrfifHhDYfgasaacH8akY=wiFfYdH8Gipec8Eeeu0xXdbba9frFj0=OqFfea0dXdd9vqai=hGuQ8kuc9pgc9s8qqaq=dirpe0xb9q8qiLsFr0=vr0=vr0dc8meaabaqaciaacaGaaeqabaqabeGadaaakeaacqWGWbaCdaahaaWcbeqaaiabcIcaOiabdohaZjabcMcaPaaakiabg2da9maalaaabaWaaabmaeaacqWGjbqscqGGOaakiiGacqWF3oaAdaqhaaWcbaGaemOyaigabaGaeiikaGIaem4CamNaeiykaKcaaaqaaiabdkgaIjabg2da9iabigdaXaqaaiabdkeacbqdcqGHris5aOGaeyyzImRae83TdG2aaWbaaSqabeaacqGGOaakcqWGZbWCcqGGPaqkaaGccqGGPaqkaeaacqWGcbGqaaGaeiOla4caaa@4AA5@

## Justification

Our justification is based on extreme cases to make the proofs tractable. However, this requirement is not very critical. Our simulation and real data analysis show that even with moderate *g*_0 _value, the pseudo-global *p*-value, *p*^(*s*)^, starts to increase when our procedure has removed most of the significant genes. Besides, it jumps up very fast to reach the point that *p*^(*s*) ^will be larger than threshold *β *and stop removing genes. Let *g *be the total number of sub-hypotheses considered. Given *g*, let ***d ***= (*d*_1_,..., *d*_*g*_) denote the vector containing the true values of *μ*_*j*_, *j *= 1,..., *g *for each sub-hypothesis; and *R*(*g*, ***d***) be the number of ***Y***^(*j*) ^removed using procedure ℘
 MathType@MTEF@5@5@+=feaafiart1ev1aaatCvAUfKttLearuWrP9MDH5MBPbIqV92AaeXatLxBI9gBaebbnrfifHhDYfgasaacH8akY=wiFfYdH8Gipec8Eeeu0xXdbba9frFj0=OqFfea0dXdd9vqai=hGuQ8kuc9pgc9s8qqaq=dirpe0xb9q8qiLsFr0=vr0=vr0dc8meaabaqaciaacaGaaeqabaqabeGadaaakeaacqGHyeYWaaa@2E34@. Without loss of generality, we assume *d*_(1) _≤ ... ≤ d(g−g0)
 MathType@MTEF@5@5@+=feaafiart1ev1aaatCvAUfKttLearuWrP9MDH5MBPbIqV92AaeXatLxBI9gBaebbnrfifHhDYfgasaacH8akY=wiFfYdH8Gipec8Eeeu0xXdbba9frFj0=OqFfea0dXdd9vqai=hGuQ8kuc9pgc9s8qqaq=dirpe0xb9q8qiLsFr0=vr0=vr0dc8meaabaqaciaacaGaaeqabaqabeGadaaakeaacqWGKbazdaWgaaWcbaGaeiikaGIaem4zaCMaeyOeI0Iaem4zaC2aaSbaaWqaaiabicdaWaqabaWccqGGPaqkaeqaaaaa@349C@ < 0 and *d*_(*j*) _= 0 for *j *= *g *- *g*_0 _+ 1,..., *g*. We have

E℘[R(g,d)]≤g−g0+E℘[R(g0,0)],     (9)
 MathType@MTEF@5@5@+=feaafiart1ev1aaatCvAUfKttLearuWrP9MDH5MBPbIqV92AaeXatLxBI9gBaebbnrfifHhDYfgasaacH8akY=wiFfYdH8Gipec8Eeeu0xXdbba9frFj0=OqFfea0dXdd9vqai=hGuQ8kuc9pgc9s8qqaq=dirpe0xb9q8qiLsFr0=vr0=vr0dc8meaabaqaciaacaGaaeqabaqabeGadaaakeaacqWGfbqrdaWgaaWcbaGaeyigHmmabeaakiabcUfaBjabdkfasjabcIcaOiabdEgaNjabcYcaSGqadiab=rgaKjabcMcaPiabc2faDjabgsMiJkabdEgaNjabgkHiTiabdEgaNnaaBaaaleaacqaIWaamaeqaaOGaey4kaSIaemyrau0aaSbaaSqaaiabgIriddqabaGccqGGBbWwcqWGsbGucqGGOaakcqWGNbWzdaWgaaWcbaGaeGimaadabeaakiabcYcaSGqabiab+bdaWiabcMcaPiabc2faDjabcYcaSiaaxMaacaWLjaWaaeWaaeaacqaI5aqoaiaawIcacaGLPaaaaaa@50E8@

and the equality holds when d(g−g0)→−∞
 MathType@MTEF@5@5@+=feaafiart1ev1aaatCvAUfKttLearuWrP9MDH5MBPbIqV92AaeXatLxBI9gBaebbnrfifHhDYfgasaacH8akY=wiFfYdH8Gipec8Eeeu0xXdbba9frFj0=OqFfea0dXdd9vqai=hGuQ8kuc9pgc9s8qqaq=dirpe0xb9q8qiLsFr0=vr0=vr0dc8meaabaqaciaacaGaaeqabaqabeGadaaakeaacqWGKbazdaWgaaWcbaGaeiikaGIaem4zaCMaeyOeI0Iaem4zaC2aaSbaaWqaaiabicdaWaqabaWccqGGPaqkaeqaaOGaeyOKH4QaeyOeI0IaeyOhIukaaa@38F1@. The equality affirms that the sub-sample ***Y***^(*j*) ^with extreme small *d*_*j *_< 0 is identified and removed from the component of *η*. Actually, when the gene expression difference is far away from zero, it will be identified by any reasonable multiple testing procedure. Hence, in this extreme case, we only focus on E℘[R(g0,0)]
 MathType@MTEF@5@5@+=feaafiart1ev1aaatCvAUfKttLearuWrP9MDH5MBPbIqV92AaeXatLxBI9gBaebbnrfifHhDYfgasaacH8akY=wiFfYdH8Gipec8Eeeu0xXdbba9frFj0=OqFfea0dXdd9vqai=hGuQ8kuc9pgc9s8qqaq=dirpe0xb9q8qiLsFr0=vr0=vr0dc8meaabaqaciaacaGaaeqabaqabeGadaaakeaacqWGfbqrdaWgaaWcbaGaeyigHmmabeaakiabcUfaBjabdkfasjabcIcaOiabdEgaNnaaBaaaleaacqaIWaamaeqaaOGaeiilaWccbeGae8hmaadcbaGae4xkaKIaeiyxa0faaa@392B@ which is based on the remaining *g*_0 _samples that have null distributions. Our goal is reduced to finding an upper bond of E℘[R(g0,0)]
 MathType@MTEF@5@5@+=feaafiart1ev1aaatCvAUfKttLearuWrP9MDH5MBPbIqV92AaeXatLxBI9gBaebbnrfifHhDYfgasaacH8akY=wiFfYdH8Gipec8Eeeu0xXdbba9frFj0=OqFfea0dXdd9vqai=hGuQ8kuc9pgc9s8qqaq=dirpe0xb9q8qiLsFr0=vr0=vr0dc8meaabaqaciaacaGaaeqabaqabeGadaaakeaacqWGfbqrdaWgaaWcbaGaeyigHmmabeaakiabcUfaBjabdkfasjabcIcaOiabdEgaNnaaBaaaleaacqaIWaamaeqaaOGaeiilaWccbeGae8hmaadcbaGae4xkaKIaeiyxa0faaa@392B@, so that the expected value of g^0
 MathType@MTEF@5@5@+=feaafiart1ev1aaatCvAUfKttLearuWrP9MDH5MBPbIqV92AaeXatLxBI9gBaebbnrfifHhDYfgasaacH8akY=wiFfYdH8Gipec8Eeeu0xXdbba9frFj0=OqFfea0dXdd9vqai=hGuQ8kuc9pgc9s8qqaq=dirpe0xb9q8qiLsFr0=vr0=vr0dc8meaabaqaciaacaGaaeqabaqabeGadaaakeaacuWGNbWzgaqcamaaBaaaleaacqaIWaamaeqaaaaa@2F2D@ can be bounded (below) by *g*_0_.

### J.1 Monotone in Pseudo-global *p*-values

The key feature of our algorithm is that the pseudo-global *p*-values are monotone increasing. We can be more precise by showing that

Pr[p(s)≤p(s+1)|p(s)]→1 as sg0→0.     (10)
 MathType@MTEF@5@5@+=feaafiart1ev1aaatCvAUfKttLearuWrP9MDH5MBPbIqV92AaeXatLxBI9gBaebbnrfifHhDYfgasaacH8akY=wiFfYdH8Gipec8Eeeu0xXdbba9frFj0=OqFfea0dXdd9vqai=hGuQ8kuc9pgc9s8qqaq=dirpe0xb9q8qiLsFr0=vr0=vr0dc8meaabaqaciaacaGaaeqabaqabeGadaaakeaatCvAUfeBSjuyZL2yd9gzLbvyNv2CaeHbwvMCKfMBHbaceiGaa8huaiaa=jhadaWadaqaaiabdchaWnaaCaaaleqabaGaeiikaGIaem4CamNaeiykaKcaaOGaeyizImQaemiCaa3aaWbaaSqabeaacqGGOaakcqWGZbWCcqGHRaWkcqaIXaqmcqGGPaqkaaGcdaabbaqaaiabdchaWnaaDaaaleaaaeaacqGGOaakcqWGZbWCcqGGPaqkaaaakiaawEa7aaGaay5waiaaw2faaiabgkziUkabigdaXiabbccaGiabbggaHjabbohaZjabbccaGmaalaaabaGaem4CamhabaGaem4zaC2aaSbaaSqaaiabicdaWaqabaaaaOGaeyOKH4QaeGimaaJaeiOla4IaaCzcaiaaxMaadaqadaqaaiabigdaXiabicdaWaGaayjkaiaawMcaaaaa@61D2@

Romano (Section 2)[23] has proved that the distribution of ηb(s)
 MathType@MTEF@5@5@+=feaafiart1ev1aaatCvAUfKttLearuWrP9MDH5MBPbIqV92AaeXatLxBI9gBaebbnrfifHhDYfgasaacH8akY=wiFfYdH8Gipec8Eeeu0xXdbba9frFj0=OqFfea0dXdd9vqai=hGuQ8kuc9pgc9s8qqaq=dirpe0xb9q8qiLsFr0=vr0=vr0dc8meaabaqaciaacaGaaeqabaqabeGadaaakeaaiiGacqWF3oaAdaqhaaWcbaGaemOyaigabaGaeiikaGIaem4CamNaeiykaKcaaaaa@32FA@ can be approximated by Gaussian distribution using the Central Limit theorem. Therefore, if we let the mean and standard deviation of reference samples ηb(s)
 MathType@MTEF@5@5@+=feaafiart1ev1aaatCvAUfKttLearuWrP9MDH5MBPbIqV92AaeXatLxBI9gBaebbnrfifHhDYfgasaacH8akY=wiFfYdH8Gipec8Eeeu0xXdbba9frFj0=OqFfea0dXdd9vqai=hGuQ8kuc9pgc9s8qqaq=dirpe0xb9q8qiLsFr0=vr0=vr0dc8meaabaqaciaacaGaaeqabaqabeGadaaakeaaiiGacqWF3oaAdaqhaaWcbaGaemOyaigabaGaeiikaGIaem4CamNaeiykaKcaaaaa@32FA@ (*b *= 1,..., *B*) be *μ*^(*s*) ^and *σ*^(*s*)^, respectively, and use *Z *to denote the standard Gaussian random variable with distribution function Φ, then the pseudo-global *p*-value *p*^(*s*) ^can be expressed as

p(s)=∑b=1BI(ηb(s)≥η(s)B=˙Pr[Z>η(s)−μ(s)σ(s)]=Pr[Z>C(s)],     (11)
 MathType@MTEF@5@5@+=feaafiart1ev1aaatCvAUfKttLearuWrP9MDH5MBPbIqV92AaeXatLxBI9gBaebbnrfifHhDYfgasaacH8akY=wiFfYdH8Gipec8Eeeu0xXdbba9frFj0=OqFfea0dXdd9vqai=hGuQ8kuc9pgc9s8qqaq=dirpe0xb9q8qiLsFr0=vr0=vr0dc8meaabaqaciaacaGaaeqabaqabeGadaaakqaaeeqaaiabdchaWnaaCaaaleqabaGaeiikaGIaem4CamNaeiykaKcaaOGaeyypa0ZaaSaaaeaadaaeWaqaaiabdMeajjabcIcaOGGaciab=D7aOnaaDaaaleaacqWGIbGyaeaacqGGOaakcqWGZbWCcqGGPaqkaaGccqGHLjYScqWF3oaAdaahaaWcbeqaaiabcIcaOiabdohaZjabcMcaPaaaaeaacqWGIbGycqGH9aqpcqaIXaqmaeaacqWGcbGqa0GaeyyeIuoaaOqaaiabdkeacbaaaeaacuGH9aqpgaGaamXvP5wqSXMqHnxAJn0BKvguHDwzZbqegyvzYrwyUfgaiqGacaGFqbGaa4NCamaadmaabaGaa4Nwaiaa+5dadaWcaaqaaiab=D7aOnaaCaaaleqabaGaeiikaGIaem4CamNaeiykaKcaaOGaeyOeI0Iae8hVd02aaWbaaSqabeaacqGGOaakcqWGZbWCcqGGPaqkaaaakeaacqWFdpWCdaahaaWcbeqaaiabcIcaOiabdohaZjabcMcaPaaaaaaakiaawUfacaGLDbaaaeaacqGH9aqpcaGFqbGaa4NCamaadmaabaGaa4Nwaiaa+5dacaGFdbWaaWbaaSqabeaacqGGOaakcqWGZbWCcqGGPaqkaaaakiaawUfacaGLDbaacqGGSaalcaWLjaGaaCzcamaabmaabaGaeGymaeJaeGymaedacaGLOaGaayzkaaaaaaa@79EF@

where *C*^(*s*) ^= Φ^-1^(1 - *p*^(*s*)^) is *p*^(*s*)^th upper percentile of the standard Gaussian distribution. Hence, the relation between *η*^(*s*) ^and *C*^(*s*) ^is

*η*^(*s*) ^= *μ*^(*s*) ^+ *σ*^(*s*) ^*C *^(*s*)^.

Similarly, for the *μ*^(*s*+1) ^and *σ*^(*s*+1) ^as the mean and standard deviation of reference samples ηb(s+1)
 MathType@MTEF@5@5@+=feaafiart1ev1aaatCvAUfKttLearuWrP9MDH5MBPbIqV92AaeXatLxBI9gBaebbnrfifHhDYfgasaacH8akY=wiFfYdH8Gipec8Eeeu0xXdbba9frFj0=OqFfea0dXdd9vqai=hGuQ8kuc9pgc9s8qqaq=dirpe0xb9q8qiLsFr0=vr0=vr0dc8meaabaqaciaacaGaaeqabaqabeGadaaakeaaiiGacqWF3oaAdaqhaaWcbaGaemOyaigabaGaeiikaGIaem4CamNaey4kaSIaeGymaeJaeiykaKcaaaaa@34CC@(*b *= 1,..., *B*), we repeat the same approximation method to express *p*^(*s*+1) ^as

p(s+1)     (12)=∑b=1BI(ηb(s+1)≥η(s+1))B=˙Pr[Z>η(s+1)−μ(s+1)σ(s+1)]=Pr[Z>(η(s+1)+ℏ(s+1))−(μ(s+1)+ℏ(s+1))σ(s+1)]=Pr[Z>η(s)−(μ(s+1)+ℏ(s+1))σ(s+1)]=Pr[Z>μ(s)+σ(s)C(s)−(μ(s+1)+ℏ(s+1))σ(s+1)]=Pr[Z>σ(s)σ(s+1)C(s)+μ(s)−μ(s+1)−ℏ(s+1)σ(s+1)].     (13)
 MathType@MTEF@5@5@+=feaafiart1ev1aaatCvAUfKttLearuWrP9MDH5MBPbIqV92AaeXatLxBI9gBaebbnrfifHhDYfgasaacH8akY=wiFfYdH8Gipec8Eeeu0xXdbba9frFj0=OqFfea0dXdd9vqai=hGuQ8kuc9pgc9s8qqaq=dirpe0xb9q8qiLsFr0=vr0=vr0dc8meaabaqaciaacaGaaeqabaqabeGadaaakeaafaqaaeacdaaaaaqaaiabdchaWnaaCaaaleqabaGaeiikaGIaem4CamNaey4kaSIaeGymaeJaeiykaKcaaaGcbaaabaGaaCzcaiaaxMaadaqadaqaaiabigdaXiabikdaYaGaayjkaiaawMcaaaqaaiabg2da9aqaamaalaaabaWaaabmaeaacqWGjbqscqGGOaakiiGacqWF3oaAdaqhaaWcbaGaemOyaigabaGaeiikaGIaem4CamNaey4kaSIaeGymaeJaeiykaKcaaaqaaiabdkgaIjabg2da9iabigdaXaqaaiabdkeacbqdcqGHris5aOGaeyyzImRae83TdG2aaWbaaSqabeaacqGGOaakcqWGZbWCcqGHRaWkcqaIXaqmcqGGPaqkaaGccqGGPaqkaeaaieGacqGFcbGqaaaabaaabaGafyypa0JbaiaaaeaacqGFqbaucqGFYbGCdaWadaqaaiab+PfaAjab+5da+maalaaabaGae83TdG2aaWbaaSqabeaacqGGOaakcqWGZbWCcqGHRaWkcqaIXaqmcqGGPaqkaaGccqGHsislcqWF8oqBdaahaaWcbeqaaiabcIcaOiabdohaZjabgUcaRiabigdaXiabcMcaPaaaaOqaaiab=n8aZnaaCaaaleqabaGaeiikaGIaem4CamNaey4kaSIaeGymaeJaeiykaKcaaaaaaOGaay5waiaaw2faaaqaaaqaaiabg2da9aqaaiab+bfaqjab+jhaYnaadmaabaGae4NwaOLae4Npa4ZaaSaaaeaacqGGOaakcqWF3oaAdaahaaWcbeqaaiabcIcaOiabdohaZjabgUcaRiabigdaXiabcMcaPaaakiabgUcaRiabl+qiOnaaBaaaleaacqGGOaakcqWGZbWCcqGHRaWkcqaIXaqmcqGGPaqkaeqaaOGaeiykaKIaeyOeI0IaeiikaGIae8hVd02aaWbaaSqabeaacqGGOaakcqWGZbWCcqGHRaWkcqaIXaqmcqGGPaqkaaGccqGHRaWkcqWIpecAdaWgaaWcbaGaeiikaGIaem4CamNaey4kaSIaeGymaeJaeiykaKcabeaakiabcMcaPaqaaiab=n8aZnaaCaaaleqabaGaeiikaGIaem4CamNaey4kaSIaeGymaeJaeiykaKcaaaaaaOGaay5waiaaw2faaaqaaaqaaiabg2da9aqaaiab+bfaqjab+jhaYnaadmaabaGae4NwaOLae4Npa4ZaaSaaaeaacqWF3oaAdaahaaWcbeqaaiabcIcaOiabdohaZjabcMcaPaaakiabgkHiTiabcIcaOiab=X7aTnaaCaaaleqabaGaeiikaGIaem4CamNaey4kaSIaeGymaeJaeiykaKcaaOGaey4kaSIaeS4dHG2aaSbaaSqaaiabcIcaOiabdohaZjabgUcaRiabigdaXiabcMcaPaqabaGccqGGPaqkaeaacqWFdpWCdaahaaWcbeqaaiabcIcaOiabdohaZjabgUcaRiabigdaXiabcMcaPaaaaaaakiaawUfacaGLDbaaaeaaaeaacqGH9aqpaeaacqGFqbaucqGFYbGCdaWadaqaaiab+PfaAjab+5da+maalaaabaGae8hVd02aaWbaaSqabeaacqGGOaakcqWGZbWCcqGGPaqkaaGccqGHRaWkcqWFdpWCdaahaaWcbeqaaiabcIcaOiabdohaZjabcMcaPaaakiabdoeadnaaCaaaleqabaGaeiikaGIaem4CamNaeiykaKcaaOGaeyOeI0IaeiikaGIae8hVd02aaWbaaSqabeaacqGGOaakcqWGZbWCcqGHRaWkcqaIXaqmcqGGPaqkaaGccqGHRaWkcqWIpecAdaWgaaWcbaGaeiikaGIaem4CamNaey4kaSIaeGymaeJaeiykaKcabeaakiabcMcaPaqaaiab=n8aZnaaCaaaleqabaGaeiikaGIaem4CamNaey4kaSIaeGymaeJaeiykaKcaaaaaaOGaay5waiaaw2faaaqaaaqaaiabg2da9aqaaiab+bfaqjab+jhaYnaadeaabaGaemOwaOLaeyOpa4ZaaSaaaeaacqWFdpWCdaahaaWcbeqaaiabcIcaOiabdohaZjabcMcaPaaaaOqaaiab=n8aZnaaCaaaleqabaGaeiikaGIaem4CamNaey4kaSIaeGymaeJaeiykaKcaaaaakiabdoeadnaaCaaaleqabaGaeiikaGIaem4CamNaeiykaKcaaOGaey4kaScacaGLBbaaaeaaaeaaaeGabaqeciaaxMaadaWacaqaamaalaaabaGae8hVd02aaWbaaSqabeaacqGGOaakcqWGZbWCcqGGPaqkaaGccqGHsislcqWF8oqBdaahaaWcbeqaaiabcIcaOiabdohaZjabgUcaRiabigdaXiabcMcaPaaakiabgkHiTiabl+qiOnaaBaaaleaacqGGOaakcqWGZbWCcqGHRaWkcqaIXaqmcqGGPaqkaeqaaaGcbaGae83Wdm3aaWbaaSqabeaacqGGOaakcqWGZbWCcqGHRaWkcqaIXaqmcqGGPaqkaaaaaaGccaGLDbaacqGGUaGlaeaacaWLjaGaaCzcamaabmaabaGaeGymaeJaeG4mamdacaGLOaGaayzkaaaaaaaa@2E2C@

Comparing equations (11) and (13), we observe that to prove *p*^(*s*) ^≤ *p*^(*s*+1) ^with probability 1 asymptotically when *p*^(*s*) ^is given is equivalent to prove that

σ(s)σ(s+1)C(s)+μ(s)−μ(s+1)−ℏ(s+1)σ(s+1)<C(s)     (14)
 MathType@MTEF@5@5@+=feaafiart1ev1aaatCvAUfKttLearuWrP9MDH5MBPbIqV92AaeXatLxBI9gBaebbnrfifHhDYfgasaacH8akY=wiFfYdH8Gipec8Eeeu0xXdbba9frFj0=OqFfea0dXdd9vqai=hGuQ8kuc9pgc9s8qqaq=dirpe0xb9q8qiLsFr0=vr0=vr0dc8meaabaqaciaacaGaaeqabaqabeGadaaakeaadaWcaaqaaGGaciab=n8aZnaaCaaaleqabaGaeiikaGIaem4CamNaeiykaKcaaaGcbaGae83Wdm3aaWbaaSqabeaacqGGOaakcqWGZbWCcqGHRaWkcqaIXaqmcqGGPaqkaaaaaOGaem4qam0aaWbaaSqabeaacqGGOaakcqWGZbWCcqGGPaqkaaGccqGHRaWkdaWcaaqaaiab=X7aTnaaCaaaleqabaGaeiikaGIaem4CamNaeiykaKcaaOGaeyOeI0Iae8hVd02aaWbaaSqabeaacqGGOaakcqWGZbWCcqGHRaWkcqaIXaqmcqGGPaqkaaGccqGHsislcqWIpecAdaWgaaWcbaGaeiikaGIaem4CamNaey4kaSIaeGymaeJaeiykaKcabeaaaOqaaiab=n8aZnaaCaaaleqabaGaeiikaGIaem4CamNaey4kaSIaeGymaeJaeiykaKcaaaaakiabgYda8iabdoeadnaaCaaaleqabaGaeiikaGIaem4CamNaeiykaKcaaOGaaCzcaiaaxMaadaqadaqaaiabigdaXiabisda0aGaayjkaiaawMcaaaaa@6335@

with probability 1 asymptotically when *C*^(*s*) ^is given.

As *g*_0 _→ ∞, the sample deviations *σ*^(*s*) ^and *σ*^(*s*+1) ^are almost identical so that σ(s)σ(s+1)
 MathType@MTEF@5@5@+=feaafiart1ev1aaatCvAUfKttLearuWrP9MDH5MBPbIqV92AaeXatLxBI9gBaebbnrfifHhDYfgasaacH8akY=wiFfYdH8Gipec8Eeeu0xXdbba9frFj0=OqFfea0dXdd9vqai=hGuQ8kuc9pgc9s8qqaq=dirpe0xb9q8qiLsFr0=vr0=vr0dc8meaabaqaciaacaGaaeqabaqabeGadaaakeaadaWcaaqaaGGaciab=n8aZnaaCaaaleqabaGaeiikaGIaem4CamNaeiykaKcaaaGcbaGae83Wdm3aaWbaaSqabeaacqGGOaakcqWGZbWCcqGHRaWkcqaIXaqmcqGGPaqkaaaaaaaa@38BC@*C*^(*s*) ^→ *C*^(*s*)^. Next, using table (8) again, we can express *μ*^(*s*) ^- *μ*^(*s*+1) ^as

∑b=1Bℏ(pb(s+1))B.
 MathType@MTEF@5@5@+=feaafiart1ev1aaatCvAUfKttLearuWrP9MDH5MBPbIqV92AaeXatLxBI9gBaebbnrfifHhDYfgasaacH8akY=wiFfYdH8Gipec8Eeeu0xXdbba9frFj0=OqFfea0dXdd9vqai=hGuQ8kuc9pgc9s8qqaq=dirpe0xb9q8qiLsFr0=vr0=vr0dc8meaabaqaciaacaGaaeqabaqabeGadaaakeaadaWcaaqaamaaqadabaGaeS4dHGMaeiikaGIaemiCaa3aaSbaaSqaaiabdkgaIjabcIcaOiabdohaZjabgUcaRiabigdaXiabcMcaPaqabaGccqGGPaqkaSqaaiabdkgaIjabg2da9iabigdaXaqaaiabdkeacbqdcqGHris5aaGcbaGaemOqaieaaiabc6caUaaa@3FC3@

The distribution of the reference set *p*_1(*s*+1)_,..., *p*_*B*(*s*+1) _converges to the uniform (0, 1) distribution since the distribution of the permuted test statistics, which are corresponding to these *p*-values, converges to a Normal distribution using Theorem 2.1 of Romano's work. Therefore, if we define uniform (0, 1) random variable as *U*, ∑b=1Bℏ(pb(s+1))B
 MathType@MTEF@5@5@+=feaafiart1ev1aaatCvAUfKttLearuWrP9MDH5MBPbIqV92AaeXatLxBI9gBaebbnrfifHhDYfgasaacH8akY=wiFfYdH8Gipec8Eeeu0xXdbba9frFj0=OqFfea0dXdd9vqai=hGuQ8kuc9pgc9s8qqaq=dirpe0xb9q8qiLsFr0=vr0=vr0dc8meaabaqaciaacaGaaeqabaqabeGadaaakeaadaWcaaqaamaaqadabaGaeS4dHGMaeiikaGIaemiCaa3aaSbaaSqaaiabdkgaIjabcIcaOiabdohaZjabgUcaRiabigdaXiabcMcaPaqabaGccqGGPaqkaSqaaiabdkgaIjabg2da9iabigdaXaqaaiabdkeacbqdcqGHris5aaGcbaGaemOqaieaaaaa@3EDF@ converges in probability to *E*[*ħ*(*U*)] by the Law of Large Number. Hence, to prove equation (14) we only need to prove that

*Pr*[*ħ*(*p*_(*s*+1)_) ≥ *E*[*ħ*(*U*)]] → 1.     (15)

Assume, without loss of generality, that *p*_1_,..., pg0
 MathType@MTEF@5@5@+=feaafiart1ev1aaatCvAUfKttLearuWrP9MDH5MBPbIqV92AaeXatLxBI9gBaebbnrfifHhDYfgasaacH8akY=wiFfYdH8Gipec8Eeeu0xXdbba9frFj0=OqFfea0dXdd9vqai=hGuQ8kuc9pgc9s8qqaq=dirpe0xb9q8qiLsFr0=vr0=vr0dc8meaabaqaciaacaGaaeqabaqabeGadaaakeaacqWGWbaCdaWgaaWcbaGaem4zaC2aaSbaaWqaaiabicdaWaqabaaaleqaaaaa@30BE@ correspond to the true null. To prove equation (15), we first observe that, since *p*_1_,... ,pg0
 MathType@MTEF@5@5@+=feaafiart1ev1aaatCvAUfKttLearuWrP9MDH5MBPbIqV92AaeXatLxBI9gBaebbnrfifHhDYfgasaacH8akY=wiFfYdH8Gipec8Eeeu0xXdbba9frFj0=OqFfea0dXdd9vqai=hGuQ8kuc9pgc9s8qqaq=dirpe0xb9q8qiLsFr0=vr0=vr0dc8meaabaqaciaacaGaaeqabaqabeGadaaakeaacqWGWbaCdaWgaaWcbaGaem4zaC2aaSbaaWqaaiabicdaWaqabaaaleqaaaaa@30BE@ are independent uniform random variables, *P*_(*s*+1) _is the (*s *+ 1)/*g*_0_-th quantile of the uniform (0, 1) distribution. By defining ζs+1,g0=(s+1)/g0
 MathType@MTEF@5@5@+=feaafiart1ev1aaatCvAUfKttLearuWrP9MDH5MBPbIqV92AaeXatLxBI9gBaebbnrfifHhDYfgasaacH8akY=wiFfYdH8Gipec8Eeeu0xXdbba9frFj0=OqFfea0dXdd9vqai=hGuQ8kuc9pgc9s8qqaq=dirpe0xb9q8qiLsFr0=vr0=vr0dc8meaabaqaciaacaGaaeqabaqabeGadaaakeaaiiGacqWF2oGEdaWgaaWcbaGaem4CamNaey4kaSIaeGymaeJaeiilaWIaem4zaC2aaSbaaWqaaiabicdaWaqabaaaleqaaOGaeyypa0JaeiikaGIaem4CamNaey4kaSIaeGymaeJaeiykaKIaei4la8Iaem4zaC2aaSbaaSqaaiabicdaWaqabaaaaa@3E94@ and using the distribution of sample quantile [24], we have

g0(p(s+1)−ςs+1,g0)→N(0,ςs+1,g0(1−ςs+1,g0)).
 MathType@MTEF@5@5@+=feaafiart1ev1aaatCvAUfKttLearuWrP9MDH5MBPbIqV92AaeXatLxBI9gBaebbnrfifHhDYfgasaacH8akY=wiFfYdH8Gipec8Eeeu0xXdbba9frFj0=OqFfea0dXdd9vqai=hGuQ8kuc9pgc9s8qqaq=dirpe0xb9q8qiLsFr0=vr0=vr0dc8meaabaqaciaacaGaaeqabaqabeGadaaakeaadaGcaaqaaiabdEgaNnaaBaaaleaacqaIWaamaeqaaaqabaGccqGGOaakcqWGWbaCdaWgaaWcbaGaeiikaGIaem4CamNaey4kaSIaeGymaeJaeiykaKcabeaakiabgkHiTGGaciab=j8awnaaBaaaleaacqWGZbWCcqGHRaWkcqaIXaqmcqGGSaalcqWGNbWzdaWgaaadbaGaeGimaadabeaaaSqabaGccqGGPaqkcqGHsgIRcqWGobGtcqGGOaakcqaIWaamcqGGSaalcqWFcpGvdaWgaaWcbaGaem4CamNaey4kaSIaeGymaeJaeiilaWIaem4zaC2aaSbaaWqaaiabicdaWaqabaaaleqaaOGaeiikaGIaeGymaeJaeyOeI0Iae8NWdy1aaSbaaSqaaiabdohaZbqabaGccqGHRaWkcqaIXaqmcqGGSaalcqWGNbWzdaWgaaWcbaGaeGimaadabeaakiabcMcaPiabcMcaPiabc6caUaaa@5CD3@

Since the first derivative of *ħ *exists, we can use the delta method to have

g0(ℏ(p(s+1))−ℏ(ςs+1,g0))→N(0,σ*2),
 MathType@MTEF@5@5@+=feaafiart1ev1aaatCvAUfKttLearuWrP9MDH5MBPbIqV92AaeXatLxBI9gBaebbnrfifHhDYfgasaacH8akY=wiFfYdH8Gipec8Eeeu0xXdbba9frFj0=OqFfea0dXdd9vqai=hGuQ8kuc9pgc9s8qqaq=dirpe0xb9q8qiLsFr0=vr0=vr0dc8meaabaqaciaacaGaaeqabaqabeGadaaakeaadaGcaaqaaiabdEgaNnaaBaaaleaacqaIWaamaeqaaaqabaGccqGGOaakcqWIpecAcqGGOaakcqWGWbaCdaWgaaWcbaGaeiikaGIaem4CamNaey4kaSIaeGymaeJaeiykaKcabeaakiabcMcaPiabgkHiTiabl+qiOjabcIcaOGGaciab=j8awnaaBaaaleaacqWGZbWCcqGHRaWkcqaIXaqmcqGGSaalcqWGNbWzdaWgaaadbaGaeGimaadabeaaaSqabaGccqGGPaqkcqGGPaqkcqGHsgIRcqWGobGtcqGGOaakcqaIWaamcqGGSaalcqWFdpWCcqGGQaGkdaahaaWcbeqaaiabikdaYaaakiabcMcaPiabcYcaSaaa@51D3@

where σ*=ςs+1,g0(1−ςs+1,g0)[ℏ'(ςs+1,g0)]2
 MathType@MTEF@5@5@+=feaafiart1ev1aaatCvAUfKttLearuWrP9MDH5MBPbIqV92AaeXatLxBI9gBaebbnrfifHhDYfgasaacH8akY=wiFfYdH8Gipec8Eeeu0xXdbba9frFj0=OqFfea0dXdd9vqai=hGuQ8kuc9pgc9s8qqaq=dirpe0xb9q8qiLsFr0=vr0=vr0dc8meaabaqaciaacaGaaeqabaqabeGadaaakeaaiiGacqWFdpWCdaahaaWcbeqaaiabcQcaQaaakiabg2da9maakaaabaGae8NWdy1aaSbaaSqaaiabdohaZjabgUcaRiabigdaXiabcYcaSiabdEgaNnaaBaaameaacqaIWaamaeqaaaWcbeaakiabcIcaOiabigdaXiabgkHiTiab=j8awnaaBaaaleaacqWGZbWCcqGHRaWkcqaIXaqmcqGGSaalcqWGNbWzdaWgaaadbaGaeGimaadabeaaaSqabaGccqGGPaqkcqGGBbWwcqWIpecAcqGGNaWjcqGGOaakcqWFcpGvdaWgaaWcbaGaem4CamNaey4kaSIaeGymaeJaeiilaWIaem4zaC2aaSbaaWqaaiabicdaWaqabaaaleqaaOGaeiykaKIaeiyxa01aaWbaaSqabeaacqaIYaGmaaaabeaaaaa@54DA@ represents the asymptotical standard error of *ħ*(*p*_(*s*+1)_).

Finally, equation (15) can be proven as,

Pr[ℏ(p(s+1))<E[ℏ(U)]]=Pr[ℏ(p(s+1))−ℏ(ςs+1,g0)σ∗     <E[ℏ(U)]−ℏ(ςs+1,g0)σ∗]=˙Φ(E[ℏ(U)]−ℏ(ςs+1,g0)σ∗)
 MathType@MTEF@5@5@+=feaafiart1ev1aaatCvAUfKttLearuWrP9MDH5MBPbIqV92AaeXatLxBI9gBaebbnrfifHhDYfgasaacH8akY=wiFfYdH8Gipec8Eeeu0xXdbba9frFj0=OqFfea0dXdd9vqai=hGuQ8kuc9pgc9s8qqaq=dirpe0xb9q8qiLsFr0=vr0=vr0dc8meaabaqaciaacaGaaeqabaqabeGadaaakqGabeabaWvaaGwaaq4aaa5abaWexLMBbXgBcf2CPn2qVrwzqf2zLnharyGvLjhzH5wyaGabciaa=bfacaWFYbGaei4waSLaeS4dHGMaeiikaGIaemiCaa3aaSbaaSqaaiabcIcaOiabdohaZjabgUcaRiabigdaXiabcMcaPaqabaGccqGGPaqkcqGH8aapcqWGfbqrcqGGBbWwcqWIpecAcqGGOaakcqWGvbqvcqGGPaqkcqGGDbqxcqGGDbqxaeaacaWLjaGaeyypa0Jaa8huaiaa=jhadaWabaqaamaalaaabaGaeS4dHGMaeiikaGIaemiCaa3aaSbaaSqaaiabcIcaOiabdohaZjabgUcaRiabigdaXiabcMcaPaqabaGccqGGPaqkcqGHsislcqWIpecAcqGGOaakiiGacqGFcpGvdaWgaaWcbaGaem4CamNaey4kaSIaeGymaeJaeiilaWIaem4zaC2aaSbaaWqaaiabicdaWaqabaaaleqaaOGaeiykaKcabaGae43Wdm3aaWbaaSqabeaaiiaacqqFxiIkaaaaaaGccaGLBbaaaeaacaWLjaGaaCzcamaadiaabiqaaqMacaWLjaGaeyipaWZaaSaaaeaacqWGfbqrcqGGBbWwcqWIpecAcqGGOaakcqWGvbqvcqGGPaqkcqGGDbqxcqGHsislcqWIpecAcqGGOaakcqGFcpGvdaWgaaWcbaGaem4CamNaey4kaSIaeGymaeJaeiilaWIaem4zaC2aaSbaaWqaaiabicdaWaqabaaaleqaaOGaeiykaKcabaGae43Wdm3aaWbaaSqabeaacqqFxiIkaaaaaaGccaGLDbaaaeaacaWLjaGafyypa0JbaiaacqqHMoGrdaqadaqaamaalaaabaGaemyrauKaei4waSLaeS4dHGMaeiikaGIaemyvauLaeiykaKIaeiyxa0LaeyOeI0IaeS4dHGMaeiikaGIae4NWdy1aaSbaaSqaaiabdohaZjabgUcaRiabigdaXiabcYcaSiabdEgaNnaaBaaameaacqaIWaamaeqaaaWcbeaakiabcMcaPaqaaiab+n8aZnaaCaaaleqabaGae03fIOcaaaaaaOGaayjkaiaawMcaaaaaaa@A31E@

which converges to 0 because *E*[*ħ*(*U*)] is finite and ℏ(ςs+1,g0)→∞ as ςs+1,g0=(s+1)/g0→0
 MathType@MTEF@5@5@+=feaafiart1ev1aaatCvAUfKttLearuWrP9MDH5MBPbIqV92AaeXatLxBI9gBaebbnrfifHhDYfgasaacH8akY=wiFfYdH8Gipec8Eeeu0xXdbba9frFj0=OqFfea0dXdd9vqai=hGuQ8kuc9pgc9s8qqaq=dirpe0xb9q8qiLsFr0=vr0=vr0dc8meaabaqaciaacaGaaeqabaqabeGadaaakeaacqWIpecAcqGGOaakiiGacqWFcpGvdaWgaaWcbaGaem4CamNaey4kaSIaeGymaeJaeiilaWIaem4zaC2aaSbaaWqaaiabicdaWaqabaaaleqaaOGaeiykaKIaeyOKH4QaeyOhIuQaeeiiaaIaeeyyaeMaee4CamNaeeiiaaIae8NWdy1aaSbaaSqaaiabdohaZjabgUcaRiabigdaXiabcYcaSiabdEgaNnaaBaaameaacqaIWaamaeqaaaWcbeaakiabg2da9iabcIcaOiabdohaZjabgUcaRiabigdaXiabcMcaPiabc+caViabdEgaNnaaBaaaleaacqaIWaamaeqaaOGaeyOKH4QaeGimaadaaa@5452@.

### J.2 Determining the threshold *β *by (3)

First, we recall that, as *p*^(0) ^is the global *p*-value based on all null data, it is uniformly distributed. That is,

*Pr*[*p*^(0) ^≤ *β*] = *β*.

We have proved that the pseudo-global *p*-values, *p*^(*s*)^, are monotone increasing when *s*/*g*_0 _→ 0. Therefore, there exists *m *such that *p*^(0) ^≤ *p*^(1) ^≤ ... ≤ *p*^(*m*)^. Furthermore, for all *s *≤ *m*,

*Pr*[*p*^(*s*) ^≤ *β*|*p*^(*j*) ^≤ *β*, *j *= 0, ..., *s *- 1] ≤ *β*.

The purpose here is to derive equation (3) for any *β *within (0,1) using the above inequality. We start with the number of genes removed by our procedure. Observe that

E℘[R(g0,0)]=∑k=0g0−1kPr[R(g0,0)=k]=∑k=1g0−1kPr[p(0)≤α,p(1)≤β,…,p(k−1)≤β,p(k)>β].
 MathType@MTEF@5@5@+=feaafiart1ev1aaatCvAUfKttLearuWrP9MDH5MBPbIqV92AaeXatLxBI9gBaebbnrfifHhDYfgasaacH8akY=wiFfYdH8Gipec8Eeeu0xXdbba9frFj0=OqFfea0dXdd9vqai=hGuQ8kuc9pgc9s8qqaq=dirpe0xb9q8qiLsFr0=vr0=vr0dc8meaabaqaciaacaGaaeqabaqabeGadaaakqGabeqaaavabaGaemyrau0aaSbaaSqaaiabgIriddqabaGccqGGBbWwcqWGsbGucqGGOaakcqWGNbWzdaWgaaWcbaGaeGimaadabeaakiabcYcaSGqabiab=bdaWGqaaiab+LcaPiab+1faDbqaaiaaxMaafaqaaeWacaaabaGaeyypa0dabiqaaazadaaeWbqaaiabdUgaRHqaciab9bfaqjab9jhaYjabcUfaBjabdkfasjabcIcaOiabdEgaNnaaBaaaleaacqaIWaamaeqaaOGaeiilaWIae8hmaaJaeiykaKIaeyypa0Jaem4AaSMaeiyxa0faleaacqWGRbWAcqGH9aqpcqaIWaamaeaacqWGNbWzdaWgaaadbaGaeGimaadabeaaliabgkHiTiabigdaXaqdcqGHris5aaGcbaGaeyypa0dabiqaaqyadaaeWbqaaiabdUgaRjab9bfaqjab9jhaYbWcbaGaem4AaSMaeyypa0JaeGymaedabaGaem4zaC2aaSbaaWqaaiabicdaWaqabaWccqGHsislcqaIXaqma0GaeyyeIuoakmaadeaabaGaemiCaa3aaWbaaSqabeaacqGGOaakcqaIWaamcqGGPaqkaaGccqGHKjYOiiGacqaFXoqycqGGSaalcqWGWbaCdaahaaWcbeqaaiabcIcaOiabigdaXiabcMcaPaaakiabgsMiJkab8j7aIjabcYcaSiablAciljabcYcaSiabdchaWnaaCaaaleqabaGaeiikaGIaem4AaSMaeyOeI0IaeGymaeJaeiykaKcaaOGaeyizImQaeWNSdiMaeWhlaWcacaGLBbaaaeaaaeaadaWacaqaceaaGfGaemiCaa3aaWbaaSqabeaacqGGOaakcqWGRbWAcqGGPaqkaaGccqGH+aGpcqaFYoGyaiaaw2faaiabc6caUaaaaaaa@8D66@

In addition,

Pr[p(0)≤β,p(1)≤β,…,p(k−1)≤β,p(k)>β]=Pr[p(0)≤β]{∏s=1k−1Pr[p(s)≤β|p(l)≤β,l=0,…,s−1]}Pr[p(k)>β|p(i)≤β,i=0,…,k−1]≤ββmin(k−1,m)1=βkm
 MathType@MTEF@5@5@+=feaafiart1ev1aaatCvAUfKttLearuWrP9MDH5MBPbIqV92AaeXatLxBI9gBaebbnrfifHhDYfgasaacH8akY=wiFfYdH8Gipec8Eeeu0xXdbba9frFj0=OqFfea0dXdd9vqai=hGuQ8kuc9pgc9s8qqaq=dirpe0xb9q8qiLsFr0=vr0=vr0dc8meaabaqaciaacaGaaeqabaqabeGadaaakqGabeqaaWvabaWexLMBbXgBcf2CPn2qVrwzqf2zLnharyGvLjhzH5wyaGabciaa=bfacaWFYbGaei4waSLaemiCaa3aaWbaaSqabeaacqGGOaakcqaIWaamcqGGPaqkaaGccqGHKjYOiiGacqGFYoGycqGGSaalcqWGWbaCdaahaaWcbeqaaiabcIcaOiabigdaXiabcMcaPaaakiabgsMiJkab+j7aIjabcYcaSiablAciljabcYcaSiabdchaWnaaCaaaleqabaGaeiikaGIaem4AaSMaeyOeI0IaeGymaeJaeiykaKcaaOGaeyizImQae4NSdiMaeiilaWIaemiCaa3aaWbaaSqabeaacqGGOaakcqWGRbWAcqGGPaqkaaGccqGH+aGpcqGFYoGycqGGDbqxaeaacaWLjaqbaeaabuGaaaaabaGaeyypa0dabaGaa8huaiaa=jhadaWadaqaaiabdchaWnaaCaaaleqabaGaeiikaGIaeGimaaJaeiykaKcaaOGaeyizImQae4NSdigacaGLBbGaayzxaaaabaaabaWaaiWaaeaadaqeWbqaaiaa=bfacaWFYbWaamWaaeaacaWFWbWaaWbaaSqabeaacqGGOaakcqWGZbWCcqGGPaqkaaGccqGHKjYOcqGFYoGydaabbaqaaiabdchaWnaaCaaaleqabaGaeiikaGIaemiBaWMaeiykaKcaaOGaeyizImQae4NSdiMae4hlaWIaemiBaWMaeyypa0JaeGimaaJaeiilaWIaeSOjGSKaeiilaWIaem4CamNaeyOeI0IaeGymaedacaGLhWoaaiaawUfacaGLDbaaaSqaaiabdohaZjabg2da9iabigdaXaqaaiabdUgaRjabgkHiTiabigdaXaqdcqGHpis1aaGccaGL7bGaayzFaaaabaaabaGaa8huaiaa=jhadaWadaqaaiabdchaWnaaCaaaleqabaGaeiikaGIaem4AaSMaeiykaKcaaOGaeyOpa4Jae4NSdi2aaqqaaeaacqWGWbaCdaahaaWcbeqaaiabcIcaOiabdMgaPjabcMcaPaaaaOGaay5bSdGaeyizImQae4NSdiMaeiilaWIaemyAaKMaeyypa0JaeGimaaJaeiilaWIaeSOjGSKaeiilaWIaem4AaSMaeyOeI0IaeGymaedacaGLBbGaayzxaaaabaGaeyizImkabaGae4NSdiMae4NSdi2aaWbaaSqabeaacqqGTbqBcqqGPbqAcqqGUbGBcqqGOaakcqWGRbWAcqGHsislcqaIXaqmcqGGSaalcqWGTbqBcqGGPaqkaaGccqaIXaqmaeaacqGH9aqpaeaacqGFYoGydaahaaWcbeqaaiabdUgaRnaaBaaameaacqWGTbqBaeqaaaaaaaaaaaa@CE42@

where *k*_*m *_= min(*k*, *m *+ 1). Hence, we can find an upper bound of the expected number of genes removed, E℘[R(g0,0)]
 MathType@MTEF@5@5@+=feaafiart1ev1aaatCvAUfKttLearuWrP9MDH5MBPbIqV92AaeXatLxBI9gBaebbnrfifHhDYfgasaacH8akY=wiFfYdH8Gipec8Eeeu0xXdbba9frFj0=OqFfea0dXdd9vqai=hGuQ8kuc9pgc9s8qqaq=dirpe0xb9q8qiLsFr0=vr0=vr0dc8meaabaqaciaacaGaaeqabaqabeGadaaakeaacqWGfbqrdaWgaaWcbaGaeyigHmmabeaakiabcUfaBjabdkfasjabcIcaOiabdEgaNnaaBaaaleaacqaIWaamaeqaaOGaeiilaWccbeGae8hmaadcbaGae4xkaKIaeiyxa0faaa@392B@, which is

E℘[R(g0,0)]≤∑k=1g0−1kβkm=β(1−β)2(1−βm+1)−(m+1)βm+2/(1−β)+(g0−m−2)βm+1≈β(1−β)2 as m is large.     (16)
 MathType@MTEF@5@5@+=feaafiart1ev1aaatCvAUfKttLearuWrP9MDH5MBPbIqV92AaeXatLxBI9gBaebbnrfifHhDYfgasaacH8akY=wiFfYdH8Gipec8Eeeu0xXdbba9frFj0=OqFfea0dXdd9vqai=hGuQ8kuc9pgc9s8qqaq=dirpe0xb9q8qiLsFr0=vr0=vr0dc8meaabaqaciaacaGaaeqabaqabeGadaaakqGabeGaaasaaGwabaGaemyrau0aaSbaaSqaaiabgIriddqabaGccqGGBbWwcqWGsbGucqGGOaakcqWGNbWzdaWgaaWcbaGaeGimaadabeaakiabcYcaSGqabiab=bdaWGqaaiab+LcaPiab+1faDbqaaiaaxMaafaqaaeabdaaaaeaacqGHKjYOaeaadaaeWbqaaiabdUgaRHGaciab9j7aInaaCaaaleqabaGaem4AaS2aaSbaaWqaaiabd2gaTbqabaaaaaWcbaGaem4AaSMaeyypa0JaeGymaedabaGaem4zaC2aaSbaaWqaaiabicdaWaqabaWccqGHsislcqaIXaqma0GaeyyeIuoaaOqaaaqaaiabg2da9aqaamaalaaabaGae0NSdigabaGaeiikaGIaeGymaeJaeyOeI0Iae0NSdiMaeiykaKYaaWbaaSqabeaacqaIYaGmaaaaaOGaeiikaGIaeGymaeJaeyOeI0Iae0NSdi2aaWbaaSqabeaacqWGTbqBcqGHRaWkcqaIXaqmaaGccqGGPaqkcqGHsislcqGGOaakcqWGTbqBcqGHRaWkcqaIXaqmcqGGPaqkcqqFYoGydaahaaWcbeqaaiabd2gaTjabgUcaRiabikdaYaaakiabc+caViabcIcaOiabigdaXiabgkHiTiab9j7aIjabcMcaPaqaaaqaaaqaaiabgUcaRiabcIcaOiabdEgaNnaaBaaaleaacqaIWaamaeqaaOGaeyOeI0IaemyBa0MaeyOeI0IaeGOmaiJaeiykaKIae0NSdi2aaWbaaSqabeaacqWGTbqBcqGHRaWkcqaIXaqmaaaakeaaaeaacqGHijYUaeaadaWcaaqaaiab9j7aIbqaaiabcIcaOiabigdaXiabgkHiTiab9j7aIjabcMcaPmaaCaaaleqabaGaeGOmaidaaaaakiabbccaGiabbggaHjabbohaZjabbccaGiabd2gaTjabbccaGiabbMgaPjabbohaZjabbccaGiabbYgaSjabbggaHjabbkhaYjabbEgaNjabbwgaLjabb6caUaqaaiaaxMaacaWLjaWaaeWaaeaacqqGXaqmcqqG2aGnaiaawIcacaGLPaaaaaaaaaa@9CFC@

Let Δ = Δ(*β*) = *β*/(1 - *β*)^2^. From equations (9) and (16), we have

E℘[R(g,d)−Δ]≤g−g0
 MathType@MTEF@5@5@+=feaafiart1ev1aaatCvAUfKttLearuWrP9MDH5MBPbIqV92AaeXatLxBI9gBaebbnrfifHhDYfgasaacH8akY=wiFfYdH8Gipec8Eeeu0xXdbba9frFj0=OqFfea0dXdd9vqai=hGuQ8kuc9pgc9s8qqaq=dirpe0xb9q8qiLsFr0=vr0=vr0dc8meaabaqaciaacaGaaeqabaqabeGadaaakeaacqWGfbqrdaWgaaWcbaGaeyigHmmabeaakiabcUfaBjabdkfasjabcIcaOiabdEgaNjabcYcaSmXvP5wqSXMqHnxAJn0BKvguHDwzZbqegyvzYrwyUfgaiqWacaWFKbGaeiykaKIaeyOeI0IaeyiLdqKaeiyxa0LaeyizImQaem4zaCMaeyOeI0Iaem4zaC2aaSbaaSqaaiabicdaWaqabaaaaa@4B44@

so that the number of true null estimate is

g^0
 MathType@MTEF@5@5@+=feaafiart1ev1aaatCvAUfKttLearuWrP9MDH5MBPbIqV92AaeXatLxBI9gBaebbnrfifHhDYfgasaacH8akY=wiFfYdH8Gipec8Eeeu0xXdbba9frFj0=OqFfea0dXdd9vqai=hGuQ8kuc9pgc9s8qqaq=dirpe0xb9q8qiLsFr0=vr0=vr0dc8meaabaqaciaacaGaaeqabaqabeGadaaakeaacuWGNbWzgaqcamaaBaaaleaacqaIWaamaeqaaaaa@2F2D@ = *g *- *R*(*g*, ***d***) + Δ

and this estimate ensures that

E℘[g^0]≥g0.
 MathType@MTEF@5@5@+=feaafiart1ev1aaatCvAUfKttLearuWrP9MDH5MBPbIqV92AaeXatLxBI9gBaebbnrfifHhDYfgasaacH8akY=wiFfYdH8Gipec8Eeeu0xXdbba9frFj0=OqFfea0dXdd9vqai=hGuQ8kuc9pgc9s8qqaq=dirpe0xb9q8qiLsFr0=vr0=vr0dc8meaabaqaciaacaGaaeqabaqabeGadaaakeaacqWGfbqrdaWgaaWcbaGaeyigHmmabeaakiabcUfaBjqbdEgaNzaajaWaaSbaaSqaaiabicdaWaqabaGccqGGDbqxcqGHLjYScqWGNbWzdaWgaaWcbaGaeGimaadabeaakiabc6caUaaa@39AD@

## Figures and Tables

**Table 1:** False discovery rate when all null hypotheses are true.

*g*	BH	aBH	Fisher	Liptak
*16*	0.047	0.047	0.048	0.048
32	0.049	0.049	0.049	0.050
64	0.049	0.049	0.049	0.049
128	0.048	0.048	0.049	0.049

**Table 2:** The means (first row) and standard errors (second row) of estimates π^0
 MathType@MTEF@5@5@+=feaafiart1ev1aaatCvAUfKttLearuWrP9MDH5MBPbIqV92AaeXatLxBI9gBaebbnrfifHhDYfgasaacH8akY=wiFfYdH8Gipec8Eeeu0xXdbba9frFj0=OqFfea0dXdd9vqai=hGuQ8kuc9pgc9s8qqaq=dirpe0xb9q8qiLsFr0=vr0=vr0dc8meaabaqaciaacaGaaeqabaqabeGadaaakeaaiiGacuWFapaCgaqcamaaBaaaleaaiiaacqGFWaamaeqaaaaa@2F9A@ based on various methods when *π*_0 _= 0.2,0.5,0.8,0.99.

True *π*_0_	0.200	0.5000	0.8000	0.9900
BUM	0.1124	0.3962	0.7288	1.0000
	0.0021	0.0024	0.0021	0.0000
SPLOSH	0.8770	0.9030	0.9343	0.9594
	0.0737	0.0592	0.0482	0.0271
LSL	0.2011	0.5015	0.8016	0.9904
	0.0005	0.0007	0.0006	0.0003
Bootstrap LSE	0.1938	0.4905	0.7863	0.9787
	0.0079	0.0135	0.0184	0.0159
QVALUE	0.1975	0.4979	0.7980	0.9871
	0.0107	0.0176	0.0242	0.0154
SEP	0.9982	0.4978	0.7982	0.9883
	0.0004	0.0078	0.0089	0.0090
mgf	-	0.4944	0.7973	0.9905
	-	0.0044	0.0055	0.0062
PRE	-	0.1712	0.6568	0.9815
	-	0.0084	0.0107	0.0066
Global-*p*	0.2005	0.5002	0.8002	0.9899
(Fisher)	0.0014	0.0012	0.0018	0.0019
Global-*p*	0.1998	0.4992	0.7978	0.9884
(Liptak)	0.0017	0.0038	0.0037	0.0027

**Table 3:** Estimates of π^0
 MathType@MTEF@5@5@+=feaafiart1ev1aaatCvAUfKttLearuWrP9MDH5MBPbIqV92AaeXatLxBI9gBaebbnrfifHhDYfgasaacH8akY=wiFfYdH8Gipec8Eeeu0xXdbba9frFj0=OqFfea0dXdd9vqai=hGuQ8kuc9pgc9s8qqaq=dirpe0xb9q8qiLsFr0=vr0=vr0dc8meaabaqaciaacaGaaeqabaqabeGadaaakeaaiiGacuWFapaCgaqcamaaBaaaleaaiiaacqGFWaamaeqaaaaa@2F9A@ based on the prostate tumor data set from the Stanford microarray database. The first four rows are estimated based on the four partitions of the whole data set while the last row the is based on the data set. The means and standard errors (5th and 6th rows) are calculated based on the estimates of the four sub-data sets.

	BUM	SPLOSH	LSL	Bootstrap LSE	QVALUE	SEP	mgf	PRE	Global-*p*
Sub-data 1	0.4011	0.4844	0.8071	0.4802	0.4662	0.5792	0.5677	0.4536	0.5583
Sub-data 2	0.5211	0.5710	0.9106	0.5797	0.5679	0.5865	0.6866	0.5727	0.6722
Sub-data 3	0.5529	0.6104	0.9302	0.6235	0.6070	0.6232	0.7120	0.6097	0.7082
Sub-data 4	0.6056	0.6627	0.9628	0.6709	0.6618	0.6760	0.7529	0.6646	0.7493
Mean	0.5202	0.5821	0.9027	0.5886	0.5758	0.6162	0.6798	0.5751	0.6702
Sd	0.0867	0.0752	0.0672	0.0813	0.0826	0.0443	0.0796	0.0894	0.0821
Whole	0.3940	0.4706	0.6598	0.3949	0.3882	0.5979	0.4706	0.2803	0.4721
